# Coated Cotton Fabrics with Antibacterial and Anti-Inflammatory Silica Nanoparticles for Improving Wound Healing

**DOI:** 10.1021/acsami.4c00383

**Published:** 2024-03-12

**Authors:** Ming Liu, Axel Guinart, Albert Granados, Carolina Gimbert-Suriñach, Ester Fernández, Roser Pleixats, Adelina Vallribera

**Affiliations:** † Department of Chemistry and Centro de Innovación en Química Avanzada (ORFEO−CINQA), Universitat Autònoma de Barcelona, Cerdanyola del Vallès, 08193 Barcelona, Spain; ‡ Departament de Biología Cel.lular, Fisiologia i Immunologia, Universitat Autònoma de Barcelona, Cerdanyola del Vallès, 08193 Barcelona, Spain

**Keywords:** silica nanoparticles, anti-inflammatory, antimicrobial, cotton wound dressing, ibuprofen, norfloxacin, amide groups, enzymatic cleavage

## Abstract

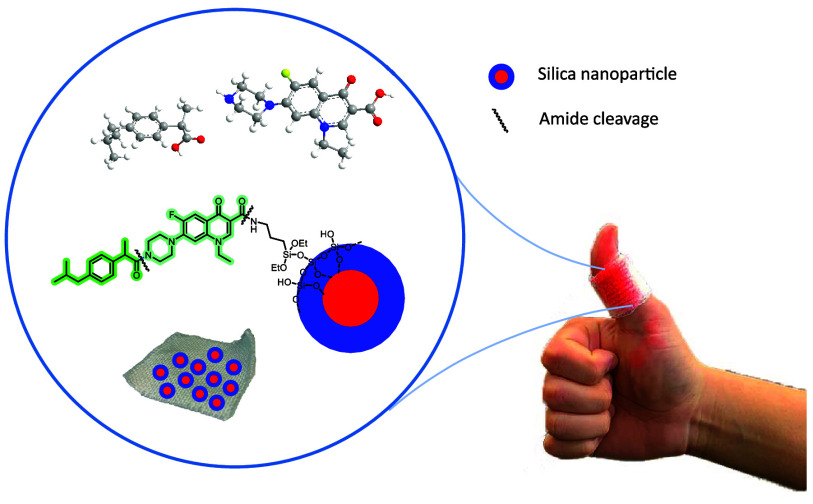

Herein, we report the preparation of bifunctional silica nanoparticles by covalent attachment of both an anti-inflammatory drug (ibuprofen) and an antibiotic (levofloxacin or norfloxacin) through amide groups. We also describe the coating of cotton fabrics with silica nanoparticles containing both ibuprofen and norfloxacin moieties linked by amide groups by using a one-step coating procedure under ultrasonic conditions. The functionalized nanoparticles and cotton fabrics have been characterized using spectroscopic and microscopic techniques. The functionalized nanoparticles and textiles have been treated with model proteases for the in situ release of the drugs by the amide bond enzymatic cleavage. Topical dermal applications in medical bandages are expected, which favor wound healing.

## 1 Introduction

The microbial infection of wounds delays wound healing and increases patient discomfort, and it can also evolve into a more serious infection. Skin wounds requiring a long time to be repaired affect human health and may constitute a major clinical problem.^1^ The systemic administration of broad-spectrum antibiotics is the most common treatment for infected wounds. However, this indiscriminate use can affect skin microbiota and prompt the proliferation of multiresistant strains. Topical administration ensures the targeted release of antimicrobial agents at the infection site, minimizing the concentration needed to control the infection and consequently reducing systemic distribution and associated toxicity.^2^

The natural low-cost fabric cotton gauze is extensively used as a medical dressing in the clinical treatment of skin wounds due to its soft nature, moisture-absorbing ability, good biocompatibility, and excellent mechanical properties. However, due to its porous structure and the abundance of hydrophilic groups on its surface, it also creates an optimal environment for the growth and survival of a large variety of bacteria, which usually results in inflammation and excessive immune response and delays wound healing. Therefore, with increasing awareness of healthcare-associated infections, there is a strong demand to develop functional medical dressings endowed with a broad spectrum of anti-inflammatory and antibacterial activities to mitigate the risk of wound infection, facilitate wound healing, and expedite cicatrization.^1−3^

Since the 1990s, natural anti-inflammatory and antibacterial agents extracted from plants have drawn growing interest in medicine. Polyphenolic compounds, including gallic acid, are particularly well-known and have been widely used for numerous applications. Taking advantage of their excellent antibacterial, antioxidant, and anti-inflammatory properties, gallic acid has been encapsulated in cotton textiles functionalized with cyclodextrin–hydroxypropyl methyl cellulose-based hydrogel. The resulting composite wound dressing was capable of releasing gallic acid in vitro, showing enhanced biological properties.^4^ Following a different strategy, Liu’s group covalently immobilized plant-derived gallic acids on an amino-rich premodified cotton gauze (after treatment with (3-aminopropyl)triethoxysilane) by reacting their phenol groups through Schiff’s base formation.^5^ Other relevant examples of functional medical gauzes include a chitosan-based hydrogel encapsulating red cabbage extract (RCE), prepared and used in therapeutic pH-sensitive wound dressing. The preparation consisted of the reaction of RCE, methacrylic -chitosan, and *N*,*N*-methylene-bis-acrylamide in the presence of potassium persulfate. The reactive mixture was then cross-linked to a piece of gauze.^6^ El-Sayed and co-workers reported the microencapsulation of anti-inflammatory and antimicrobial-rich fractions from marine macroalgae and seagrass in sodium alginate or meypro gum. They used these extracts to finish cotton fabrics by dip coating technique.^7^

Nanomaterials based on the use of abundant natural compounds are appreciated in medicinal applications and have been also used in anti-inflammatory and antibacterial agents release.^8^ For instance, mesoporous silica SBA-15 was functionalized with 3-aminopropyltrimethoxysilane (NH_2_–SBA-15) and then added into a solution of poly(vinyl alcohol) and Curcumin. Nanofibers were then fabricated by electrospinning and studied for a skin wound healing application. Curcumin, a plant-derived phytochemical hydrophobic product, is recognized for its anti-inflammatory and antimicrobial properties. The incorporation of Curcumin into the highly porous and biocompatible amine-functionalized mesoporous silica improved its solubility, facilitating sustained drug release.^9^ In 2022, Zohoori’s group extracted the keratin of hedgehog spines and doped the extract with Harmaline and Ginkgo Biloba, and later, the mixture was electrospun on the cotton surface to produce multifunctional band-aid. These nanofibers exhibited anti-inflammatory and bactericidal effects.^10^ Nitric oxide-propelled nanomotors were prepared for endotoxin removal and bacterial biofilm elimination to treat infected burn wounds. First, silica layers were deposited on Fe_3_O_4_ nanoparticles (Fe_3_O_4_ NPs) using tetraethyl orthosilicate (TEOS) and 3-aminopropyl triethoxysilane. Then, a coupling reaction of residual amino groups with 3-mercaptopropionic acid gave rise to thiolated Fe_3_O_4_ NPs. These nanoparticles (NPs) were partially coated with polydopamine (PDA) layers. Polymyxin B (PMB) was conjugated on PDA, and then, NO donors were conjugated with –SH groups to build nitric oxide-propelled nanomotors.^11^

Silver nanoparticles, one of the most used commercial antibacterial nanomaterials, have found extensive application in cotton dressings to confer effective antiseptic properties. Silver nanoparticles embedded in zwitterionic poly(carboxybetaine-*co*-dopaminemethacrylamide) copolymer have been anchored onto cotton fabrics through interaction forces that include both covalent and noncovalent bonds, which prevented leaching. In vivo wound healing assay confirmed that these Ag nanoparticles effectively inhibit the wound infection and reduce the inflammatory response.^12^ Alisir’s group described the fabrication of poly(lactic acid) nanofibers embedded with a silver diclofenac complex with 2-methylimidazole. The unique combination of diclofenac, silver(I), and 2-methylimidazole in a single product ([Ag(mim)_2_](dicl)) accelerated the healing process by endowing the wounded skin with protection against inflammation, bacterial, and fungal infections.^13^

**Figure 1:**
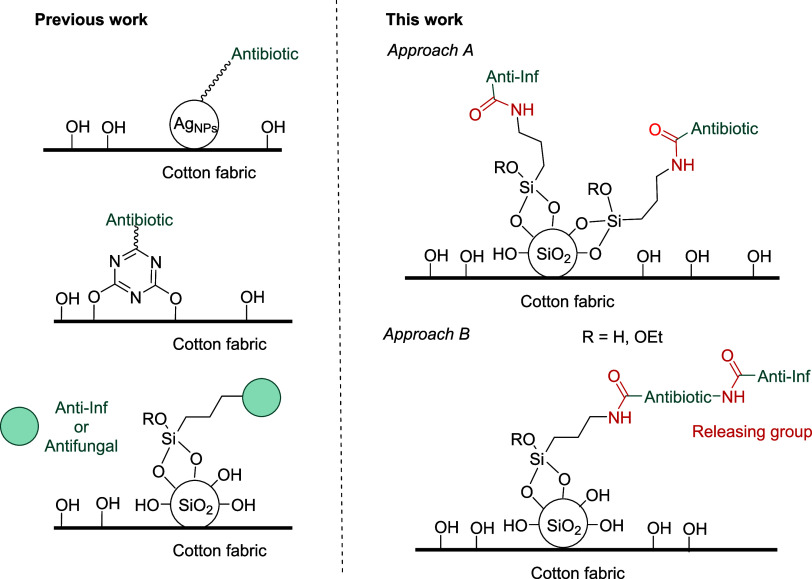
Previous work related to our program centered on the design and application of cotton fabrics for medical applications. This work: Approaches for the coating of cotton fabrics with anti-inflammatory and antibacterial silica nanoparticles.

As part of our research program centered on the design and application of cotton fabrics for medical applications (Figure 1), in 2019, we prepared silver nanoparticles (Ag NPs) with appropriate modified antibiotics, which were applied on cotton fabrics.^14^ Later, we tethered antibiotic drugs through a triazine moiety onto the surface of cotton fabrics, thus minimizing the leaching of the bioactive molecule (Figure 1). The bactericidal activity of the functionalized fabrics was demonstrated, and of note, no release of the covalent-linked antibiotic was needed.^15^

Following our program, we then prepared anti-inflammatory cotton fabrics by direct covalent attachment of anti-inflammatory (anti-inf) silylated drugs derivatives (salicylic acid, ibuprofen and diclofenac) onto the surface of cotton fabrics through an amide group.^16^ In addition, as silica nanoparticles present significant advantages in biomedical applications, we synthesized functionalized silica nanoparticles using the same drug derivatives through an amide group and used them for the coating of textiles (Figure 1, bottom left).^16^ It should be noted that in most of the published studies for drug delivery, the cargo is encapsulated and physically adsorbed into the pores, while the covalent attachment of the drug to the surface of the nanoparticle is a less used strategy. During inflammation, many leukocytes exit the blood vessels and migrate toward the injured site (chemotaxis).^17^ As a result, the phagocytic activity and the release of proteases, peroxidases, and oxygen-reactive species are increased, bolstering the breakdown and elimination of the external agents. Thus, the protease activity in a wound may be high and has been measured using an appropriate substrate, usually consisting of a peptide linked with a fluorophore or chromophore moiety through an amide bond.^18^ The detection of the chromogenic or fluorogenic moiety is thus evidence supporting that proteases can cleave amide bonds.^19,20^ In fact, when these functionalized nanoparticles and textiles are treated with proteases and leukocytes of animal origin, an in situ release of the drug takes place by the selective enzymatic cleavage of the amide bond. In contrast to the case of antibiotics reported previously, the release of the anti-inflammatory is necessary for topical cutaneous applications.

Following an analogous approach, in 2022, classical antifungal bioactive molecules were also used with success in the functionalization of silica nanoparticles (Figure 1).^21^ We described the covalent connection of silylated derivatives of the topical antifungal agent miconazole onto silica nanoparticles. Employing grafting and co-condensation procedures, we synthesized functionalized mesoporous or dense nanoparticles. The coating of cotton fabrics with these antifungal-functionalized silica nanoparticles was conducted under ultrasonic conditions, resulting in notably high effectiveness toward *Trichophyton mentagrophytes* and *Candida albicans*.^21^

Pursuing our interest in the design and application of cotton fabrics for medical applications, we planned to elaborate bifunctional silica nanoparticles with both antibiotic and anti-inflammatory properties that could be used for coating cotton gauzes or strips toward wound healing applications. Thus, herein, we describe the preparation of silica nanoparticles covalently functionalized with carboxyl-containing antibiotics and carboxyl-containing nonsteroidal anti-inflammatory drugs through amide bonds (see approach A, Figure 1). In addition, we report the preparation of silica nanoparticles derived from a silylated monomer containing both antibiotic and anti-inflammatory moieties in the same organic backbone (see approach B, Figure 1). Finally, the modification of cotton fabrics with these bifunctional silica nanoparticles is described (from Approach B) by one-step coating. We expect potential topical applications in chronic skin wounds.

## 2 Results and Discussion

**Scheme 1:**
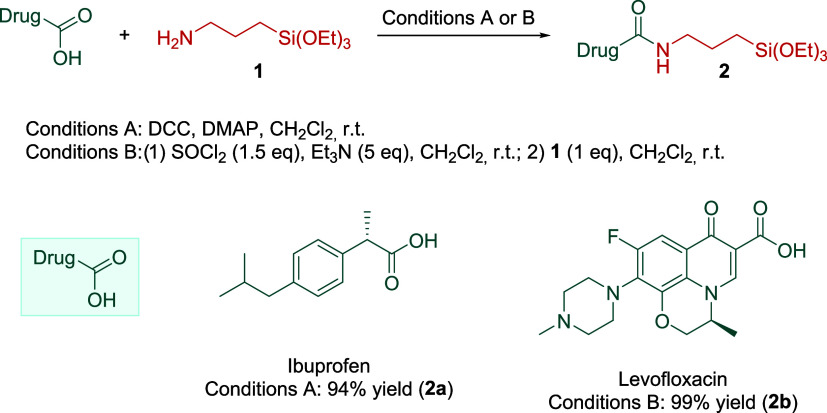
Preparation of Silylated Derivatives **2a** and **2b**

A topical nonsteroidal anti-inflammatory agent, ibuprofen, and common fluoroquinolone antibiotic drugs levofloxacin and norfloxacin were selected as model medicinally relevant molecules. A carboxylic acid group is the common moiety in all of the structures, which is necessary to form a covalent amide bond with the silylated linker (Scheme 1). In addition, the covalent grafting onto silica nanoparticles will prevent leaching of the bioactive molecule. On the other hand, the amide group is crucial for the drug release for cutaneous uses via enzymatic cleavage of the peptide bond.

The synthesis of silylated derivatives **2a**–**b** was done following two different methodologies indicated in Scheme 1. Ibuprofen was mixed with *N*,*N*′-dicyclohexylcarbodiimide (DCC), (triethoxysilyl)propylamine, and a catalytic amount of 4-(dimethylamino)pyridine (DMAP) in CH_2_Cl_2_ at room temperature affording **2a** in 50% yield. In the case of the silylated derivative of levofloxacin, we obtained product **2b** via acyl chloride synthesis followed by the addition of a stoichiometric amount of (triethoxysilyl)propylamine in 99% yield.

**Scheme 2:**
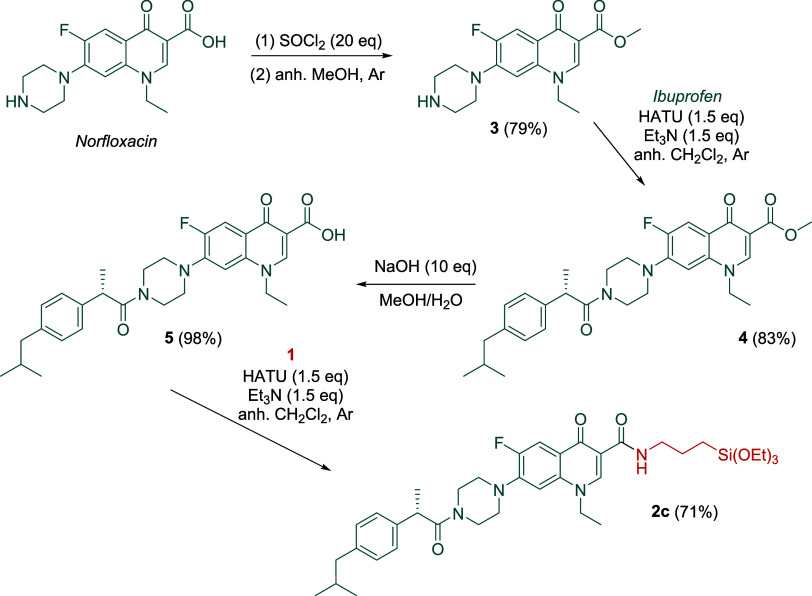
Preparation of Silylated Derivative **2c**

On the other hand, derivative **2c** was synthesized following the steps summarized in Scheme 2. In this approach B (Figure 1), norfloxacin was selected due to the nucleophilic secondary amine present in the piperazine ring, which allows covalent linking of it to the anti-inflammatory ibuprofen derivative via an amide bond (step 2, Scheme 2). First, the norfloxacin methyl ester **3** was prepared in 79% yield through the treatment of its acyl chloride derivative. Then, ibuprofen was tethered to **3** using hexafluorophosphate azabenzotriazole tetramethyl uronium (HATU) as a coupling reagent in the presence of triethylamine to form **4** in 83% yield. After basic ester hydrolysis, the coupling between acid **5** and silylated amine **1** was carried out with HATU, yielding the silylated norfloxacin derivative **2c** in 71% yield. Thus, compound **2c** was synthesized in 4 steps with a 46% overall yield.

**Scheme 3:**
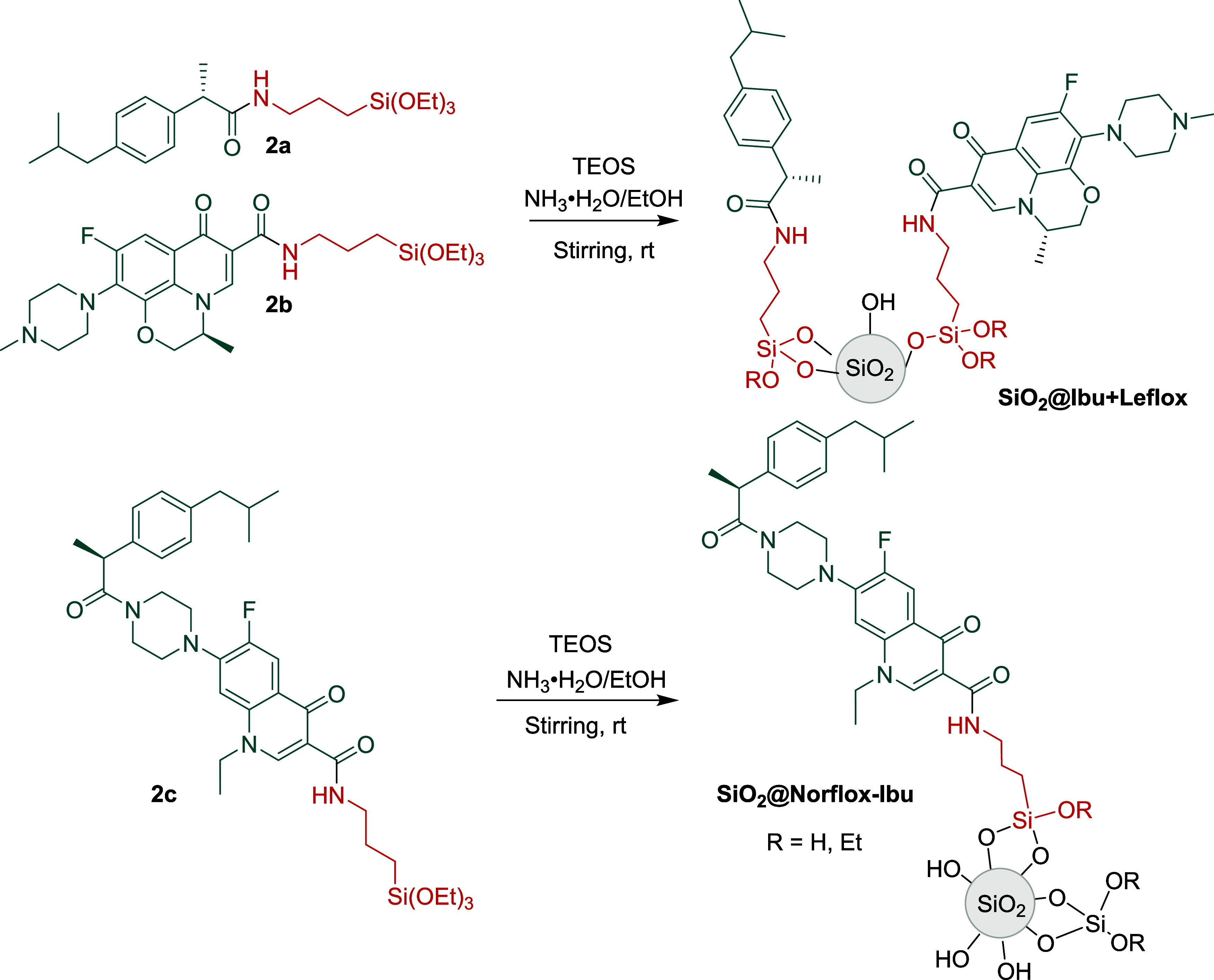
Preparation of Functionalized Silica Nanoparticles

Then, the covalent connection of antibiotics and anti-inflammatory drugs to silica nanoparticles by sol–gel condensation methods was conducted. Materials **SiO**_**2**_**@Ibu + Leflox** and **SiO**_**2**_**@Norflox-Ibu** (see Scheme 3 for the structure of these nanomaterials) were prepared by co-condensation of silylated precursors **2a**–**c** with tetraethyl orthosilicate (TEOS) using a 28% ammonium hydroxide-ethanol solution as a promoter (Scheme 3 and Table 1). For the synthesis of **SiO**_**2**_**@Ibu + Leflox**, different molar ratios TEOS/**2a/2b**/EtOH/NH_3_/H_2_O were used, maintaining a ratio TEOS/**2a** + **2b** (20:1). First, a 50:50 ratio of silylated drug derivatives **2a:2b** was assayed, obtaining modified silica nanoparticles that exhibited a low ζ-potential value, which was indicative of low stability (Table 1, entry 1). In addition, transmission electron microscopy (TEM) images showed a high level of nanoparticle aggregation, presumably due to the high concentration of the **2b**. Thus, the molar ratio of **2a**:**2b** was increased to 75:25. In this case, TEOS (16.97 mmol), the silylated ibuprofen derivative **2a** (0.63 mmol), and the silylated levofloxacin derivative **2b** (0.21 mmol) were dissolved in absolute EtOH. Then, an ammonium hydroxide-ethanol solution was added. As shown in Table 1 (entry 2), the higher ζ-potential for the new sample indicated better stability. Then, we centered our attention on approach B (Figure 1) using the same procedure for the sol–gel process but now using the monosilylated monomer **2c**. Mixtures of TEOS/**2c**/EtOH/NH_3_/H_2_O were used at three different ratios TEOS/**2c** (20:1, 30:1, and 40:1). The functionalized silica NPs showed high negative values of ζ-potential, from −65.5 to −72.3 mV, in agreement with residual deprotonated silanol groups and no protonation of the amino group, and indicative of high stability (Table 1, entries 3–5).

**Table 1:** Characterization Data of Functionalized Silica Nanoparticles

		particle size (nm)		
material	drug loading (mmol/g)^a^	TEM	DLS^b^	ζ-potential (mV)
**SiO**_**2**_**@Ibu + Leflox** (20:0.5:0.5)	0.23:0.23	/	2733	5.7
**SiO**_**2**_**@Ibu + Leflox** (20:0.75:0.25)	0.075:0.025	317 ± 22	426	–41.0
**SiO**_**2**_**@Norflox-Ibu** (20:1)	0.15	686 ± 32	836	–65.5
**SiO**_**2**_**@Norflox-Ibu** (30:1)	0.13	540 ± 30	658	–69.9
**SiO**_**2**_**@Norflox-Ibu** (40:1)	0.15	389 ± 22	486	–72.3

a Estimated from the N elemental analysis.

b Hydrodynamic diameters.

**Figure 2:**
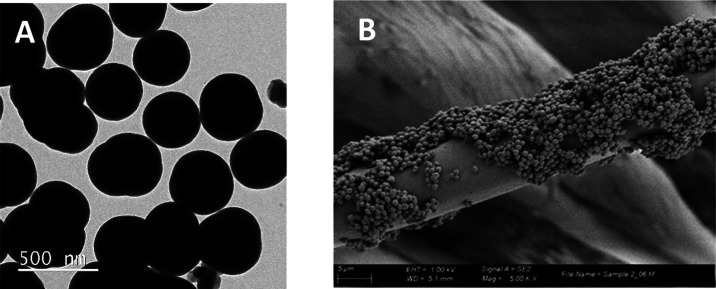
(A) TEM of **SiO**_**2**_**@Norflox-Ibu** (TEOS/**2c** 40:1). (B) SEM of **Fabric-SiO**_**2**_**@Norflox-Ibu** (TEOS/**2c** 40:1).

The TEM analysis of materials **SiO**_**2**_**@Ibu + Leflox** and **SiO**_**2**_**@Norflox-Ibu** confirmed the nanometric size and features of the functionalized nanoparticles, showing dense spherical morphologies with sizes from 317 to 686 nm (see Figure 2A for a representative image of **SiO**_**2**_**@Norflox-Ibu** (TEOS/**2c** 40:1); see also SI). Dynamic light scattering measurements (DLS) were consistent with the size observed via transmission electron microscopy (TEM) for the corresponding dried nanoparticles, considering the likely adsorption of water molecules onto the nanoparticle surface (Table 1).

**Figure 3:**
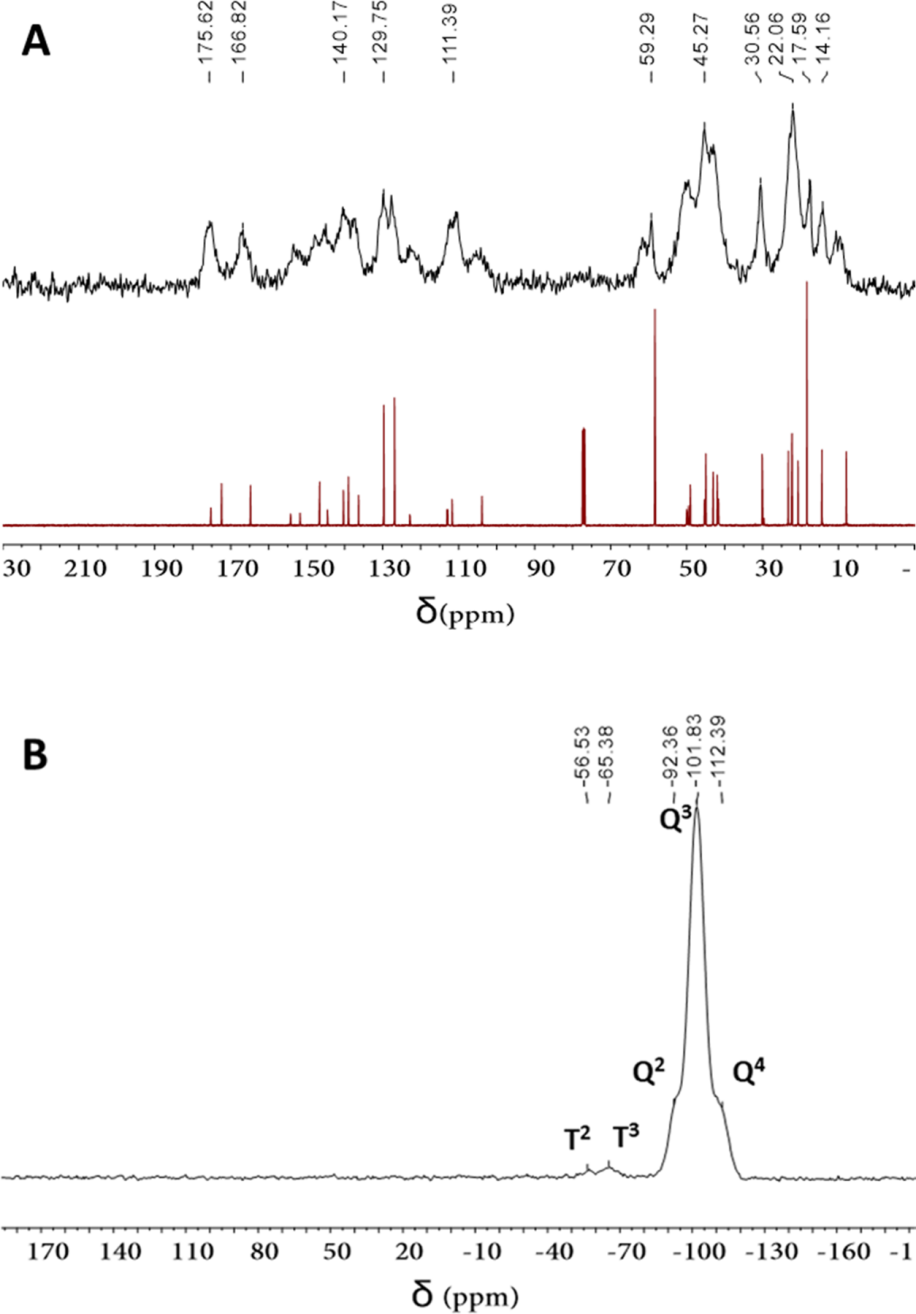
(A) ^13^C CP MAS NMR spectrum of **SiO**_**2**_**@Norflox-Ibu** (TEOS/**2c** 20:1) and ^13^C NMR spectrum of **2c**. (B) ^29^Si CP MAS NMR spectrum of **SiO**_**2**_**@Norflox-Ibu** (TEOS/**2c** 40:1).

The presence of the organic moiety in the functionalized NPs was confirmed by solid-state ^29^Si and ^13^C nuclear magnetic resonance (NMR) spectra. Thus, a clear match between the ^13^C NMR spectrum of **2c** in solution and that of **SiO**_**2**_**@Norflox-Ibu** (TEOS/**2c** 20:1) in the solid state is observed, supporting the integrity of the organic framework (Figure 3A). The ^29^Si cross-polarization magic-angle-spinning nuclear magnetic resonance (CP MAS NMR) spectrum of **SiO**_**2**_**@Norflox-Ibu** (TEOS/**2c** 40:1) showed two groups of chemical shifts: *T* units (−56.5 and −65.4 ppm) derived from organosilane **2c** and *Q* units (−92.4, −101.8, and −112.4 ppm) derived from TEOS (Figure 3B). The presence of *T* signals is also an indication that the Si–C bond of the precursors was maintained during the sol–gel co-condensation process.

The drug content in the NPs was inferred from the nitrogen elemental analysis (Table 1). For the materials **SiO**_**2**_**@Ibu + Leflox**, the given values are based on the initial amounts of silylated monomers and the estimation that **2a** and **2b** have the same condensation rate. For the nanomaterial **SiO**_**2**_**@Ibu + Leflox (20:0.75:0.25)**, the drug content found was quite low (Table 1, entry 2). The materials **SiO**_**2**_**@Norflox-Ibu** contain both drug moieties in molecule **2c**, and thus, the values correspond to the whole organic moiety in the materials.

**Figure 4:**
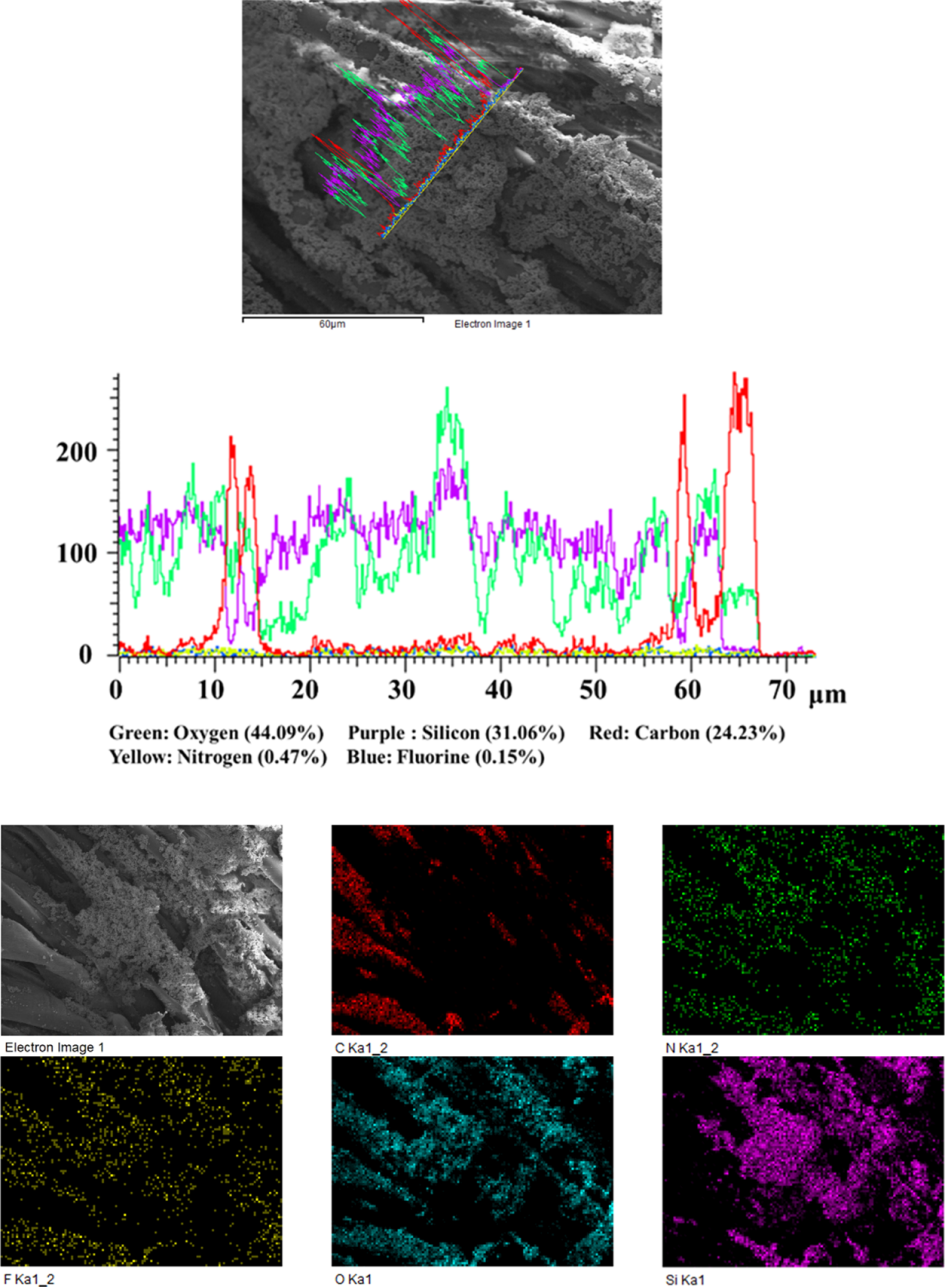
EDX linear scanning and element mapping of **Fabric-SiO**_**2**_**@Norflox-Ibu** (TEOS/**2c** 40:1).

With the drug-functionalized silica NPs in hand, we proceeded with the loading of cotton fabrics with these nanoparticles by the one-step coating method. As we have mentioned, for **SiO**_**2**_**@Ibu + Leflox**, a low proportion of antibiotic (ratio Ibu/Leflox 75:25) was required to afford stable silica nanoparticles, and the total amount of drugs in the final nanomaterial was quite low according to the elemental analysis. On the other hand, the precise amount of ibuprofen vs levofloxacin in the silica matrix cannot be estimated without assuming the same reactivity in sol–gel process for both **2a** and **2b**. For these reasons, we decided to perform the coating only with **SiO**_**2**_**@Norflox-Ibu** materials. As depicted in Table 1, the drug loading in the nanoparticles did not significantly vary with the molar ratio TEOS/**2c**. For that reason, the coating solution was obtained by hydrolysis and co-condensation of TEOS with organosilane **2c** (molar ratio 40:1) in aqueous ammonia and ethanol under stirring. Without any further isolation, the obtained milky solution was ultrasonicated for 30 min, producing a homogeneous suspension. A piece of cotton was immersed in this suspension, and the whole system was ultrasonicated for an additional half an hour. Then, after removal of the cotton fabric from the solution, it was washed with distilled water and dried at 120 °C for 1 h. By scanning electron microscopy (SEM) analysis, the presence of abundant silica nanoparticles was observed on the surface of cotton fabrics loaded with **SiO**_**2**_**@Norflox-Ibu** (TEOS/**2c** 40:1) by one-step coating (Figure 2B). The textile piece gained 10 mg after coating with NPs according to gravimetric analysis. The chemical composition of the surface of the modified fabrics was analyzed by energy-dispersive X-ray spectroscopy (EDX) (Figure 4). As expected, the EDX analysis exhibited peaks for the elements C, O, N, Si, and F. The peaks corresponding to C, N, and F indicate the presence of the organic moiety in silica nanoparticles.

The anti-inflammatory drug must be released from the fabric to be in contact with the damaged area and locally modulate the inflammation of the wounds.^16^ On the contrary, we have previously demonstrated that some fluoroquinolone derivatives covalently linked to the surface of a cotton fabric exhibited excellent antimicrobial activity for *S. aureus* being able to reduce preformed *Staphylococcus aureus* biofilms. Some experiments were carried out to demonstrate that these covalently attached microbicidal fluoroquinolone derivatives do not leach from the fabric surface, and thus, they do not contribute to the development of resistance.^15^

**Figure 5:**
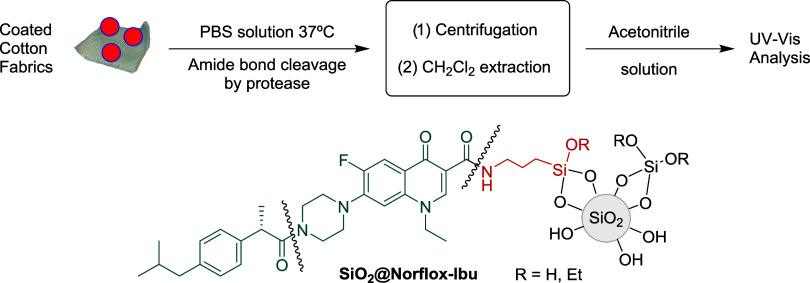
Procedure for the drug-release experiment.

With the **SiO**_**2**_**@Norflox-Ibu** (TEOS/**2c** 40:1) and **Fabric-SiO**_**2**_**@Norflox-Ibu** (TEOS/**2c** 40:1) in hand, the release of the bioactive components was studied, assaying proteases such as trypsin, papain, and proteinase K. These enzymes would produce the cleavage of the amide bonds (Figure 5), giving rise to ibuprofen and norfloxacin release. First, the detachment from nanoparticles **SiO**_**2**_**@Norflox-Ibu** (TEOS/**2c** 40:1, 20 mg) was tested (Table 2, entries 1–3). The experiments were carried out in phosphate-buffered saline (PBS) buffer (pH = 7.4, 0.2 M, 2 mL) at 37 °C under stirring for 24 h. The nanoparticles were separated by centrifugation, and the supernatant was extracted with dichloromethane. After the removal of the organic solvent, the residue was dissolved in acetonitrile. The resulting solution was analyzed by ultraviolet–visible (UV–vis) spectroscopy, and the amount of released drugs could be quantified by analyzing the intensity of the absorption peaks at λ = 220 nm for ibuprofen and λ = 285 nm for norfloxacin according to the corresponding calibration curves. The percentage of release was increased by performing a 48 h treatment (Table 2, entry 4). However, the low solubility of norfloxacin at neutral pH made the quantitative measurement inaccurate. As an alternative, we centered our attention on the qualitative detection of norfloxacin by changing the post-treatment analysis. Thus, 2 mL of glacial acetic acid was added to the mixture after protease treatment. After 30 min stirring, the mixture was centrifugated at 12,000 rpm. The supernatant was separated, diluted with water to 10 mL, analyzed by UV–vis, and compared with an acidic aqueous solution of commercial norfloxacin (see the Supporting Information). Finally, we treated a 3 cm × 3 cm cotton piece of **Fabric-SiO**_**2**_**@Norflox-Ibu** (TEOS/**2c** 40:1) with papain in PBS buffer (pH = 7.4, 0.2 M, 5 mL) at 37 °C under stirring for 48 h (Table 2, entry 5). Moreover, the supernatants of papain-treated **SiO**_**2**_**@Norflox-Ibu** (TEOS/**2c** 40:1) and **Fabric-SiO**_**2**_**@Norflox-Ibu** (TEOS/**2c** 40:1) were used to test the inhibitory effects of released norfloxacin on the growth of *S. Aureus*. Indeed, a small area of inhibition of bacterial growth could be observed (Supporting Information). Thus, this is an indication that norfloxacin has been released from the modified silica nanoparticles and fabrics upon enzymatic hydrolysis of the amide bond.

**Table 2:** Release of Drugs from Nanoparticles and Coated Fabrics

				release (%)	
entry	sample	protease	time (h)	norfloxacin	ibuprofen
1^a^	**SiO**_**2**_@Norflox-Ibu (TEOS/**2c** 40:1)	trypsin	24	0.12 ± 0.01	4.5 ± 0.3
2^a^	**SiO**_**2**_@Norflox-Ibu (TEOS/**2c** 40:1)	papain	24	0.26 ± 0.05	7.1 ± 0.4
3^a^	**SiO**_**2**_@Norflox-Ibu (TEOS/**2c** 40:1)	proteinase K	24	0.15 ± 0.05	5.8 ± 0.5
4^a^	**SiO**_**2**_@Norflox-Ibu (TEOS/**2c** 40:1)	papain	48	c	11.1 ± 0.2
5^b^	**Fabric-SiO**_**2**_@Norflox-Ibu (TEOS/**2c** 40:1)	papain	48	c	43.5 ± 2.5

a Experiments with 20 mg of NPs and 4 × 10^–4^ mmol of protease.

b Experiment with 10 mg of NPs in the coating and 1 × 10^–3^ mmol of papain.

c Qualitative detection. See text.

## 3 Conclusions

Herein, we have presented the preparation of silica nanoparticles covalently functionalized with both a carboxyl-containing antibiotic (levofloxacin) and a nonsteroidal anti-inflammatory drug (ibuprofen) through amide bonds (**SiO**_**2**_**@Ibu + Leflox**). We also synthesized silica nanoparticles derived from a monosilylated monomer bearing both carboxyl-containing antibiotic (norfloxacin) and anti-inflammatory moieties (ibuprofen) in the same molecule, linked by amide bonds (**SiO**_**2**_**@Norflox-Ibu**). All of these nanoparticles were obtained by sol–gel methodologies under basic conditions and by co-condensation of the corresponding monomers with tetraethoxysilane. These dense nanoparticles were characterized by transmission electron microscopy, dynamic light scattering, ζ-potential, solid-state ^29^Si and ^13^C NMR spectroscopy, and elemental analysis. Of note, cotton fabrics have been coated with drug-functionalized nanoparticles **SiO**_**2**_**@Norflox-Ibu** by a one-step procedure under ultrasonic conditions. The modified textiles **Fabric-SiO**_**2**_**@Norflox-Ibu** have been characterized by scanning electron microscopy, energy-dispersive X-ray spectroscopy, and element mapping. The corresponding drugs were released in situ through enzymatic cleavage of the amide bonds in **SiO**_**2**_**@Norflox-Ibu** and **Fabric-SiO**_**2**_**@Norflox-Ibu** by treatment with proteases (UV–vis analysis).

In contrast to some sophisticated procedures, our method is operationally simple. Ibuprofen and norfloxacin are classical drugs on the market that have been widely used, and therefore, all toxicity parameters have been thoroughly studied. Of note, in this study, their antimicrobial and anti-inflammatory properties are not compromised after their incorporation into the composite network because the enzymatic release does not alter their initial structure. On the other hand, silica-based nanoparticles have received special attention as a biocompatible form of silica.

Topical applications for medical gauzes provided with antibiotic and anti-inflammatory properties to prevent infection and accelerate wound healing in chronic cutaneous wounds are expected.

## 4 Experimental Section

### 4.1 General Procedure for the Preparation of **SiO**_**2**_**@Ibu + Leflox**

TEOS (2.08 g, 10.0 mmol), the silylated anti-inflammatory ibuprofen **2a** (0.25 mmol), and the silylated antibiotic levofloxacin **2b** (0.25 mmol) were dissolved in 25 mL of absolute EtOH. Then, an ethanol solution of ammonium hydroxide (6 mL of 28% NH_3_·H_2_O in 25 mL EtOH) was added. The resulting mixture was magnetically stirred (1400 rpm) at rt for 12 h, after which the nanoparticles were isolated by centrifugation (13,500 rpm) at rt and washed with ethanol until neutral pH was reached. The resulting solid was then sequentially washed with Mili-Q water and 96% ethanol and dried under a vacuum for several hours. For other molar ratio details, see the SI.

### 4.2 General Procedure for the Preparation of **SiO**_**2**_**@Norflox-Ibu**

TEOS (2.08 g, 10.0 mmol) and the silylated derivative **2c** (0.5, 0.33, and 0.25 mmol) were dissolved in 25 mL of absolute EtOH. Then, an ethanol solution of ammonium hydroxide was added (6 mL of 28% NH_3_ H_2_O in 25 mL of EtOH). The mixture was magnetically stirred (1400 rpm) at rt for 12 h. The working-up procedure was the same as described in the previous paragraph. We obtained **SiO**_**2**_**@Norflox-Ibu** with different ratios of TEOS and silylated drug **2c**. For other molar ratio details, see the SI.

### 4.3 Procedure for the Preparation of Cotton Fabrics Coated with **SiO**_**2**_**@Norflox-Ibu** (**Fabric-SiO**_**2**_**@Norflox-Ibu 40:1**)

TEOS (2.08 g, 10.0 mmol) and the silylated derivative **2c** (0.25 mmol) were dissolved in 25 mL of absolute EtOH. Then, an ethanol solution of ammonium hydroxide was added (6 mL of 28% NH_3_ H_2_O in 25 mL of EtOH). The mixture was magnetically stirred (1400 rpm) at rt for 12 h. Without any further isolation, the obtained milky solution was ultrasonicated for 30 min to produce a homogeneous suspension in which a piece of clean cotton fabric (3 cm × 3 cm) was immersed. The whole system was ultrasonicated for an additional 30 min. Then, the cotton textile was removed from the solution, washed with distilled water, and finally dried at 120 °C for 1 h.

### 4.4 Treatment of Functionalized Silica Nanoparticle **SiO**_**2**_**@Norflox-Ibu (40:1)** with Proteases for Quantitative Analysis by UV–vis

The functionalized silica nanoparticles **SiO**_**2**_**@Norflox-Ibu (40:1)** (20 mg) were dispersed in phosphate-buffered saline (PBS, pH 7.4) (2 mL) in an Eppendorf tube. Subsequently, the corresponding protease (see Table 2) was added, and the mixture was gently stirred at 37 °C for the given time (orbital shaker). The concentration of protease was 0.2 mM. Following incubation, the nanoparticles were removed by centrifugation, and the supernatant was extracted with dichloromethane (10 × 3 mL). The solvent was then evaporated under vacuum from the combined organic phases. The resulting residue was dissolved in acetonitrile, and the solution was analyzed by UV–vis (ibuprofen λ = 220 nm̧, norfloxacin λ = 285 nm). See page S33 in the SI.

### 4.5 Treatment of Functionalized Silica Nanoparticle **SiO**_**2**_**@Norflox-Ibu (40:1)** with Proteases for Qualitative Detection of Norfloxacin

The functionalized silica nanoparticles **SiO**_**2**_**@Norflox-Ibu (40:1)** (20 mg) were dispersed in phosphate-buffered saline (PBS) (2 mL) in an Eppendorf tube, the corresponding protease was added, and the mixture was gently stirred at 37 °C for 48 h (orbital shaker). The concentration of protease was 0.2 mM. Glacial acetic acid (2 mL) was added, and the mixture was stirred for 30 min. The nanoparticles were separated by centrifugation, the supernatant was diluted to 10 mL, and the aqueous solution was analyzed by UV–vis. The commercial norfloxacin aqueous solution (0.24 mmol/L) was prepared with the same amount of glacial acetic acid and was analyzed by UV–vis (λ = 285 nm). See page S32 in the SI.

### 4.6 Treatment of Cotton Fabrics Coated with Functionalized Silica Nanoparticles with Proteases

A piece of **Fabric-SiO**_**2**_**@Norflox-Ibu (40:1)** (3 cm × 3 cm) was cut into small pieces and dispersed in phosphate-buffered saline (PBS) (5 mL) in an Eppendorf tube, the corresponding protease was added, and the mixture was gently stirred at 37 °C for 48 h (orbital shaker). The concentration of protease was 0.2 mM. After the removal of cotton fabrics, the supernatant was extracted with dichloromethane (25 × 3 mL). The solvent was then evaporated under vacuum from the combined organic phases. The resulting residue was dissolved in acetonitrile, and the solution was analyzed by UV–vis.

## Supporting Information Available

General considerations, the general procedure for the preparation of **2a**–**c** as well as spectroscopic data, and characterization data of functionalized silica nanoparticles and cotton fabrics (PDF)

## References

[ref1] Pierce G. F.; Mutsoe T. A. Pharmacologic enhancement of wound healing. Annu. Rev. Med. 1995, 46, 467–481. 10.1146/annurev.med.46.1.467. 7598479

[ref2] Miraftab M. Wound Care Materials: An Overview. In Medical and Healthcare Textiles; Anand S. C.; Kennedy J. F.; Miraftab M.; Rajendran S., Eds.; Woodhead, 2010; pp 193–197.

[ref3] Chen S.; Li A.; Wang Y.; Zhang Y.; Liu X.; Ye Z.; Gao S.; Xu H.; Deng L.; Dong A.; Zhang J. Janus polyurethane sponge as an antibiofouling, antibacterial, and exudate-managing dressing for accelerated wound healing. Acta Biomater. 2023, 171, 428–439. 10.1016/j.actbio.2023.09.015. 37716478

[ref4] Pinho E.; Calhelha R. C.; Ferreira I. C. F. R.; Soares G. Cotton-hydrogel composite for improved wound healing: Antimicrobial activity and anti-inflammatory evaluation—Part 2. Polym. Adv. Technol. 2019, 30, 863–871. 10.1002/pat.4519.

[ref5] Lang S.; Chen C.; Xiang J.; Liu Y.; Li K.; Hu Q.; Liu G. Facile and robust antibacterial functionalization of medical cotton gauze with gallic acids to accelerate wound healing. Ind. Eng. Chem. Res. 2021, 60, 10225–10234. 10.1021/acs.iecr.1c01833.

[ref6] Arafa A. A.; Nada A. A.; Ibrahim A. Y.; Sajkiewicz P.; Zahran M. K.; Hakeim O. A. Preparation and characterization of smart therapeutic pH-sensitive wound dressing from red cabbage extract and chitosan hydrogel. Int. J. Biol. Macromol. 2021, 182, 1820–1831. 10.1016/j.ijbiomac.2021.05.167. 34052272

[ref7] El-Rafie H. M.; El-Rafie M. H.; AbdElsalamc H. M.; El-Sayed W. A. Antibacterial and anti-inflammatory finishing of cotton by microencapsulation using three marine organisms. Int. J. Biol. Macromol. 2016, 86, 59–64. 10.1016/j.ijbiomac.2016.01.039. 26776873

[ref8] Granados A.; Pleixats R.; Vallribera A. Recent advances on antimicrobial and anti-inflammatory cotton fabrics containing nanostructures. Molecules 2021, 26, 3008 10.3390/molecules26103008. 34070166 PMC8158507

[ref9] Rathinavel S.; Indrakumar J.; Korrapati P. S.; Dharmalingam S. Synthesis and fabrication of amine functionalized SBA-15 incorporated PVA/Curcumin nanofiber for skin wound healing application. Colloids Surf., A 2022, 637, 128185 10.1016/j.colsurfa.2021.128185.

[ref10] Monavari M.; Zohoori S.; Davodiroknabadi A. Anti-inflammatory and bactericidal effect of keratin/harmaline/ginkgo biloba electrospun nano fibres as band aid. Micro Nano Lett. 2022, 17, 210–215. 10.1049/mna2.12125.

[ref11] Peng J.; Xie S.; Huang K.; Ran P.; Wei J.; Zhang Z.; Li X. J. Nitric oxide-propelled nanomotors for bacterial biofilm elimination and endotoxin removal to treat infected burn wounds. J. Mater. Chem. B 2022, 10, 4189–4202. 10.1039/D2TB00555G. 35575383

[ref12] Xiang J.; Zhu R.; Lang S.; Yan H.; Liu G.; Peng B. Mussel-inspired immobilization of zwitterionic silver nanoparticles toward antibacterial cotton gauze for promoting wound healing. Chem. Eng. J. 2021, 409, 128291 10.1016/j.cej.2020.128291.

[ref13] Alisir S. H.; Ozdemir N.; Burgaz E.; Dege N.; Canavar Y. E. Fabrication and Antimicrobial Activity of Poly(lactic acid) Nanofibers Containing Firstly Synthesized Silver Diclofenac Complex with (2-methylimidazole) for Wound Dressing Applications. Fibers Polym. 2021, 22, 2738–2749. 10.1007/s12221-021-0166-z.

[ref14] Montagut A. M.; Granados A.; Ballesteros A.; Pleixats R.; Llagostera M.; Cortés P.; Sebastián R. M.; Vallribera A. Antibiotic protected silver nanoparticles for microbicidal cotton. Tetrahedron 2019, 75, 102–108. 10.1016/j.tet.2018.11.035.

[ref15] Montagut A. M.; Granados A.; Lazurko C.; El-Khoury A.; Suuronen E. J.; Alarcon E. I.; Sebastián R. M.; Vallribera A. Triazine Mediated Covalent Antibiotic Grafting on Cotton Fabrics as a Modular Approach for Developing Antimicrobial Barriers. Cellulose 2019, 26, 7495–7505. 10.1007/s10570-019-02584-w.

[ref16] Li H.; Granados A.; Fernández E.; Pleixats R.; Vallribera A. Anti-inflammatory cotton fabrics and silica nanoparticles with potential topical medical applications. ACS Appl. Mater. Interfaces 2020, 12, 25658–25675. 10.1021/acsami.0c06629. 32407065

[ref17] Cañedo-Dorantes L.; Cañedo-Ayala M. Skin Acute Wound Healing: A Comprehensive Review. Int. J. Inflammation 2019, 2019, 3706315 10.1155/2019/3706315. PMC6582859 31275545

[ref18] Cox S. W.; Cho K.; Eley B. M.; Smith R. E. A simple, combined fluorogenic and chromogenic method for the assay of proteases in gingival crevicular fluid. J. Periodontal Res. 1990, 25, 164–171. 10.1111/j.1600-0765.1990.tb01039.x. 2141876

[ref19] Bendicho S.; Martí G.; Hernández T.; Martín O. Determination of proteolytic activity in different milk systems. Food Chem. 2002, 79, 245–249. 10.1016/S0308-8146(02)00126-7.

[ref20] Lottenberg R.; Christensen U.; Jackson C. M.; Coleman P. L. Assay of coagulation proteases using peptide chromogenic and fluorogenic substrates. Methods Enzymol. 1981, 80, 341–361. 10.1016/S0076-6879(81)80030-4. 6210826

[ref21] Liu M.; Granados A.; Reyes-Mesa D.; Arosemena-Angulo E. L.; Calvo-Torras M. A.; Pleixats R.; Vallribera A. Silica nanostructures against fungal growth: design and preparation of antifungal cotton fabrics. Cellulose 2022, 29, 8889–8905. 10.1007/s10570-022-04726-z.

