# Supplementary material for: Coated Cotton Fabrics with Antibacterial and Anti-Inflammatory Silica Nanoparticles for Improving Wound Healing
Source: ACS Appl Mater Interfaces. 2024 Mar 12;16(12):14595–604. doi: 10.1021/acsami.4c00383 (PMC10982928; doi:10.1021/acsami.4c00383)
Supplement: Supplementary file 1 — am4c00383_si_001.pdf [file am4c00383_si_001.pdf]

Supporting Information

Coated Cotton Fabrics with Antibacterial and Anti-inflammatory Silica Nanoparticles for Improving Wound Healing

Ming Liu,[a] Axel Guinart,[a] Albert Granados,[a] Carolina Gimbert-Suriñach,[a] Ester Fernández,[b] Roser Pleixats,[a]* Adelina Vallribera[a]*

[a]Department of Chemistry and Centro de Innovación en Química Avanzada (ORFEO-CINQA), Universitat Autònoma de Barcelona, Cerdanyola del Vallès, 08193 Barcelona, Spain.

Email: adelina.vallribera@uab.es; Email: roser.pleixats@uab.cat

[b]Departament de Biologia Cel.lular, Fisiologia i Immunologia, Universitat Autònoma de Barcelona, Cerdanyola del Vallès, 08193 Barcelona, Spai*n*

Table of Contents

1. General Considerations 2

2. Preparation and description of compounds 2a-c 3

3. NMR and IR spectra 8

4. Preparation and characterization of functionalized silica nanoparticles and fabrics 16

5. Treatment of functionalized silica nanoparticle SiO_2@Norflox-Ibu (40:1) with proteases for quantitative analysis by UV-Vis 32

6. Treatment of functionalized silica nanoparticle SiO_2@Norflox-Ibu (40:1) with proteases for qualitative detection of Norfloxacin 32

7. Treatment of cotton fabrics coated with functionalized silica nanoparticles with proteases 33

8. Inhibitory tests of bacterial growth 33

1. General considerations

All chemicals were purchased from Fluorochem and Merck, and they were used without further purification, unless specified. The ^{1}H, ^{13}C and ^{19}F NMR spectra were recorded at 298 K on 360 and 400 MHz Bruker DPX or a 400 MHz Bruker Avance-III equipped with a BBFO probe with an automatic tuning. All the spectra were calibrated using the residual solvent signal ($CDCl_3$, δ_H, 7.26 and δ_C, 77.16 ppm). Chemical shift data were expressed in ppm and coupling constant (*J*) values in Hz. Multiplicity of peaks was abbreviated as s (singlet), d (doublet), t (triplet), q (quartet), dd (doublet of doublets) and m (multiplet). The ^{13}C-CPMAS was acquired from a 400 MHz Bruker NEO spectrometer at 10 KHz spinning rate, ns 15360 (18h), d1=2s. Cross-Polarization mixing time was set to 1 ms. Externally calibrated with adamantane sample (29.5 ppm). Spectra processed with a line broadening 20. The ^{29}Si-CPMAS was obtained from a 400 MHz Bruker NEO spectrometer at 10 KHz spinning rate, ns 15360 (15h), d1=1s. Cross-Polarization mixing time was set to 1ms. Externally calibrated with DSS sample (0 ppm). Spectra processed with a line broadening 40. FTIR spectroscopy was recorded with a Bruker Tensor 27 spectrometer using a Golden Gate ATR module with a diamond window. High resolution mass spectra were obtained by direct injection of the sample with electrospray techniques in Hewlett-Packard 5989A and microTOF-Q instruments, respectively. Elemental analysis of C, N, and H were performed using Flash 2000 Organic Elemental analyzer of Thermo Fisher Scientific with BBOT as an internal standard. Transmission electron microscopy (TEM) analyses were performed on a JEM-2011 Electron Microscope at 200 kV. Scanning electron microscopy (SEM), energy-dispersive X-ray spectroscopy (EDX), and element line scans mapping were taken on a Zeiss Merlin scanning electron microscope (SEM) with an INCA energy dispersive X-ray (EDX) detector from Oxford Instruments. Dynamic light scattering (DLS) and zeta potential measurements have been performed using a Zetasizer Nano ZS (Malvern Instruments) with 10 mg of silica NPs in 10 mL of Milli-Q water.

2. Preparation and description of compounds 2a-c

*Synthesis of (S)-2-(4-isobutylphenyl)-N-(3-(triethoxysilyl)propyl)propenamide, **2a***

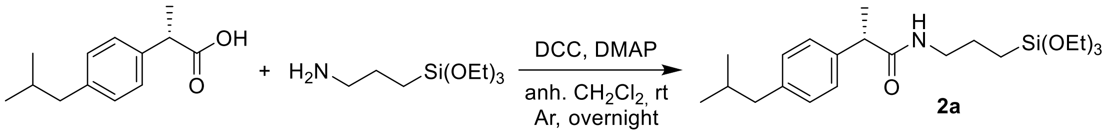

Ibuprofen (206 mg, 1 mmol) was placed in a Schlenk under argon atmosphere. Then, DMAP (6 mg, 5 mol %), DCC (206 mg, 1 mmol, 1 equiv) and dry dichloromethane (5 mL) were added, and the solution was stirred until homogenization. Then 3-(triethoxysilyl)propan-1-amine (266 mg, 1.2 mmol) was introduced by syringe. The reaction was allowed to proceed under stirring at room temperature until completion (TLC monitoring). The crude mixture was poured into water and extracted with dichloromethane. The combined organic phase was washed with brine, dried with anhydrous sodium sulphate, and evaporated under vacuum. The residue was purified by silica gel column chromatography (hexane:ethyl acetate = 3:1) to afford **2a** in 50% yield (205 mg, 0.5 mmol) ctroscopic data matches with those previously reported.[1]

^{1}H NMR (400 MHz, $CDCl_3$) δ (ppm): 7.19 (d, J = 7.9 Hz, 2H), 7.10 (d, J = 7.9 Hz, 2H), 5.62 (s, 1H), 3.51 (q, J = 7.2 Hz, 1H), 3.19 (q, J = 6.4 Hz, 2H), 2.45 (d, J = 7.2 Hz, 2H), 1.85 (dt, J = 13.5, 6.7 Hz, 1H), 1.57 – 1.47 (m, 5H), 1.19 (t, J = 7.0 Hz, 9H), 0.90 (d, J = 6.6 Hz, 6H), 0.51 (dd, J = 9.7, 6.6 Hz, 2H).

*Synthesis of (S)-9-fluoro-3-methyl-10-(4-methylpiperazin-1-yl)-7-oxo-N-(3-(triethoxysilyl)propyl)-2,3-dihydro-7H-[1,4]oxazino[2,3,4-ij]quinoline-6-carboxamide, **2b***

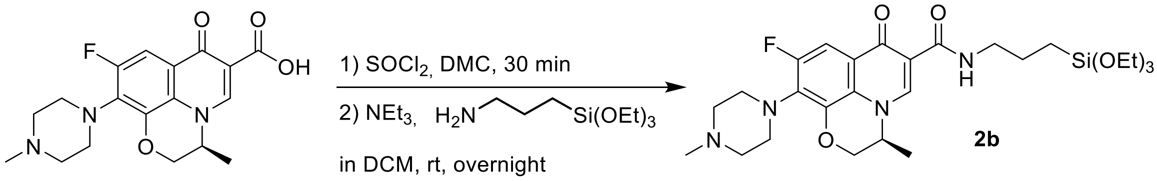

Step 1: Levofloxacin (362 mg, 1.0 mmol, 1 equiv.) was introduced in the Schlenk under argon atmosphere in 10 mL of anhydrous CH_2Cl_2. Then, 1.5 equiv. of $SOCl_2$ (179 mg, 1.5 mmol, 1.5 equiv) were added and let it react at rt under argon atmosphere for half an hour. Then the solution was evaporated to dryness to remove the solvent and excess of $SOCl_2$. The crude mixture was directly used for the next step.

[1] Li, H.; Granados, A.; Fernández, E.; Pleixats, R.; Vallribera, A. Anti-inflammatory Cotton Fabrics and Silica Nanoparticles with Potential Topical Medical Applications. *ACS Applied Materials & Interfaces* **2020,** *12,* 25658-25675.

Step 2: The remaining solid was dissolved in 5 mL of CH_2Cl_2 and triethylamine (5.0 mmol, 5 equiv) was added. Then, 1 equiv of silylated amine (101.2 mg, 1.0 mmol, 1equiv) dissolved in 5 mL of dry CH_2Cl_2 was added and led to react overnight. Distilled water (100 mL) was added and extractions with CH_2Cl_2 (4x20 mL) were carried out. The organic phase was dried over anhydrous Na_2SO_4, filtered, and evaporated to dryness to afford **2b** in 99% (563 mg, 0.99 mmol) yield.

^{1}H NMR (400 MHz, $CDCl_3$) δ (ppm): 10.01 (s, 1H), 8.64 (s, 1H), 7.71 (d, J = 12.4 Hz, 1H), 4.46 – 4.37 (m, 2H), 4.37 – 4.25 (m, 1H), 3.83 (q, J = 6.9 Hz, 6H), 3.52 – 3.32 (m, 6H), 2.59 (s, 4H), 2.40 (s, 3H), 1.76 (p, J = 7.6 Hz, 2H), 1.58 (d, J = 6.4 Hz, 3H), 1.23 (t, J = 6.9 Hz, 9H), 0.73(t, J = 8.2 Hz, 2H). **^{13}C NMR** (91 MHz, $CDCl_3$) δ (ppm):175.3, 164.9, 155.9, 155.7 (d, J = 247.5 Hz), 143.8, 139.5 (d, J = 6.6 Hz), 131.5 (d, J = 14.6 Hz), 124.3, 122.6 (d, J = 8.8 Hz), 111.2, 105.1 (d, J = 24.1 Hz), 79.0, 68.2, 50.1, 46.0, 45.8, 40.3, 38.8, 28.4, 27.6, 18.3, 8.7. **^{19}F NMR** (376 MHz, $CDCl_3$) δ (ppm):-121.2 (d, J = 12.6 Hz). **IR** (ATR) ν (cm^{-1}): 3235, 2930, 2324, 1649, 1600, 1239, 1200, 1046, 1071, 800. **HRMS** (ESI) m/z $[M+H]^+$ calcd for $C_{27}H_{42}FN_4O_6Si$ 565.2852, found: 565.2851.

<u>Synthetic route to access ***2c***</u>

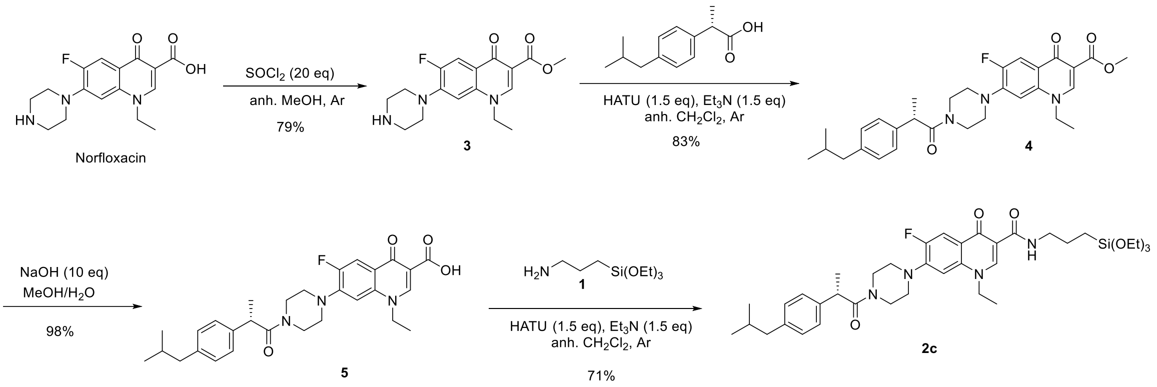

*Synthesis of methyl 1-ethyl-6-fluoro-4-oxo-7-(piperazin-1-yl)-1,4-dihydroquinoline-3-carboxylate, **3***

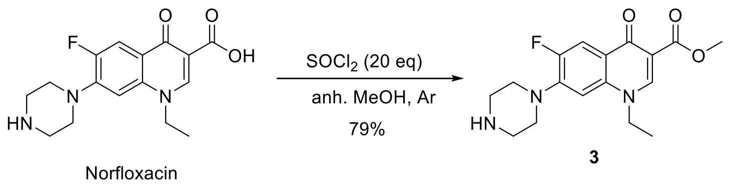

$SOCl_2$ (2.7 mL, 28.5 mmol, 20 eq) was added dropwise into a solution of norfloxacin (451 mg, 1.412 mmol, 1 eq) in 10 mL of dry methanol at 0 °C. The mixture turned to clear and was heated to reflux for 24 h. The solution was neutralized with 10% Na_2CO_3 aqueous solution. The aqueous phase was extracted with DCM:MeOH (90:10). The organic phase was dried over anhydrous Na_2SO_4, filtered, and concentrated in vacuo affording the ester **3** (371 mg, 1.11 mmol, 79%) as a

white powder. The crude was directly used for the next step. Th spectroscopic data matches with those previously reported.[2]

^{1}H NMR (400 MHz, $CDCl_3$) δ(ppm): 8.46 (s, 1H), 8.10 (d, *J* = 13.3 Hz, 1H), 6.76 (d, *J* = 6.8 Hz, 1H), 4.23 (q, *J* =7.2 Hz, 2H), 3.94 (s, 3H), 3.25 (m, 4H), 3.17 – 3.07 (m, 4H), 2.09 (bs, 1H), 1.56 (t, *J* = 7.2 Hz, 3H).

Synthesis of methyl (S)-1-ethyl-6-fluoro-7-(4-(2-(4-isobutylphenyl)propanoyl)piperazin-1-yl)-4-oxo-1,4-dihydroquinoline-3-carboxylate, ***4***

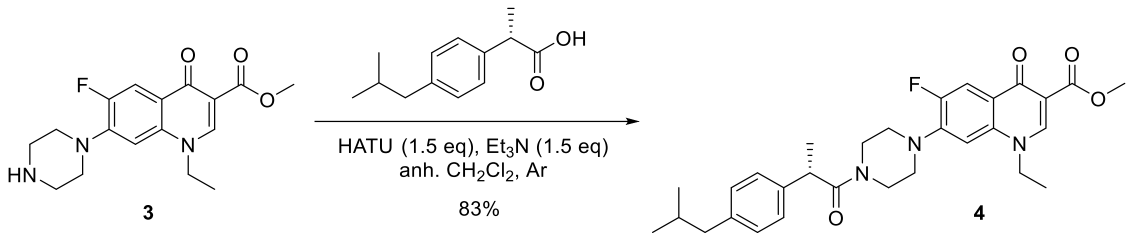

(*S*)-2-(4-isobutylphenyl)propanoic acid (200 mg, 1 mmol), Et_3N (0.2 mL, 1.4 mmol, 1.4 equivv) and HATU (0.45 g, 1.18 mmol) were dissolved in anhydrous CH_2Cl_2 (10 mL), the mixture was stirred for 30 minutes under argon atmosphere. Then the mixture was added to a solution of methyl 1-ethyl-6-fluoro-4-oxo-7-(piperazin-1-yl)-1,4-dihydroquinoline-3-carboxylate (323 mg, 0.97 mmol, 0.97 equiv) in dry CH_2Cl_2 (5 mL) at room temperature and refluxed for 48 h. The reaction was monitored by TLC. The reaction mixture was quenched with water, extracted with ethyl acetate, dried over sodium sulfate and concentrated under reduced pressure to get crude compound. The obtained crude was purified by silica gel column chromatography by using EtOAc:MeOH (90:10) as eluent to afford **4** (0.42 g, 0.805 mmol, 83 %).

MP: 96-98 °C; **^{1}H NMR** (400 MHz, $CDCl_3$) δ (ppm): 8.37 (s, 1H), 7.90 (d, *J* = 13.2 Hz, 1H), 7.17 (d, *J* = 7.8 Hz, 2H), 7.08 (d, *J* = 7.9 Hz, 2H), 6.64 (d, *J* = 6.8 Hz, 1H), 4.15 (q, *J* = 7.3 Hz, 2H), 3.91 (m, 2H), 3.83 (s, 3H), 3.75 (s, 1H), 3.65 (s, 1H), 3.52 (s, 1H), 3.11 (s, 3H), 2.68 (s, 1H), 2.42 (d, *J* = 7.1 Hz, 2H), 1.81 (m, 1H), 1.51 – 1.39 (m, 6H), 0.85 (m, 6H). **^{13}C NMR** (101 MHz, $CDCl_3$) δ (ppm): 173.8 (d, *J* = 2.0 Hz) 172.6, 165.9, 153.0 (d, *J* = 249.5 Hz), 148.2, 144.6 (d, *J* = 10.1 Hz), 140.3, 139.1, 136.1, 129.8, 127.0, 123.4 (d, *J* = 7.1 Hz), 113.4 (d, *J* = 23.2 Hz), 109.3, 104.3, 51.9, 49.8, 49.2, 45.3, 45.0, 42.9, 41.7, 30.2, 22.41, 22.37, 20.7, 14.4. **IR** (ATR) ν (cm^{-1}): 2951, 2866, 1723, 1693, 1617, 1486, 1432, 1315, 1221, 1091, 1019, 840, 803. **HRMS** (ESI) m/z $[M+H]^+$ calcd for $C_{30}H_{37}FN_3O_4$: 522.2763, found: 522.2762.

[2] Luo, X.; Wang, P. Ynonylation of Acyl Radicals by Electroinduced Homolysis of 4-Acyl-1,4-dihydropyridines. *Org. Lett.* **2021,** *23*, 4960-4965.

*Synthesis of (S)-1-ethyl-6-fluoro-7-(4-(2-(4-isobutylphenyl)propanoyl)piperazin-1-yl)-4-oxo-1,4-dihydroquinoline-3-carboxylic acid, **5***

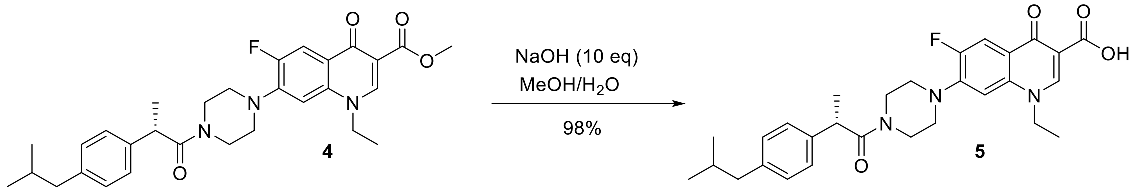

To an ice-cooled solution of ester 4 (522 mg, 1 mmol, 1 eq) in methanol (5 mL) was added 1 M NaOH aqueous solution (10 mL, 10 mmol, 10 eq). The mixture was let to stir at rt for 48 hours. Afterwards the solution was brought to pH 2-3 through the addition of 1 M HCl aqueous solution. The resulting solution was extracted with CH_2Cl_2, the organic extract was dried over anhydrous sodium sulfate and the solvent was removed in vacuo to afford a white powder. The product was recrystallized with CH_2Cl_2 and hexane to afford **5** (497.5 mg, 0.98 mmol) in 98 % yield.

MP: 182-184 °C. **^{1}H NMR** (400 MHz, $CDCl_3$) δ (ppm): 8.64 (s, 1H), 8.01 (d, *J* = 12.9 Hz, 1H), 7.15 (d, *J* = 7.9 Hz, 2H), 7.09 (d, *J* = 7.8 Hz, 2H), 6.72 (d, *J* = 6.7 Hz, 1H), 4.28 (q, *J* = 7.3 Hz, 2H), 4.06 (m, 1H), 3.88 (app q, *J* = 6.8 Hz, 1H), 3.70 (m, 1H), 3.59 (m, 2H), 3.29 (m, 1H), 3.23 – 3.05 (m, 2H), 2.66 (m, 1H), 2.43 (d, *J* = 7.2 Hz, 2H), 1.82 (m, 1H), 1.55 (t, *J* = 7.2 Hz, 3H), 1.46 (d, *J* = 6.8 Hz, 3H), 0.85 (m, 6H). **^{13}C NMR** (101 MHz, $CDCl_3$) δ (ppm): 176.9 (d, *J* = 2.0 Hz), 172.6, 167.1, 153.4 (d, *J* = 252.5 Hz), 147.2, 145.7 (d, *J* = 11.1 Hz), 140.5, 139.0, 137.1, 129.9, 127.0, 120.8 (d, *J* = 8.1 Hz), 112.8 (d, *J* = 23.2 Hz), 108.4, 104.1 (d, *J* = 3.0 Hz), 49.8, 49.5, 45.3, 45.0, 43.1, 41.6, 30.2, 22.43, 22.40, 20.8, 14.5. **IR** (ATR) ν (cm^{-1}): 2953, 2867, 1712, 1625, 1432, 1230, 1018, 805, 747. **HRMS** (ESI) m/z $[M+H]^+$ calcd for $C_{29}H_{35}FN_3O_4$: 508.2606, found: 508.2599. **AE**: calcd: 68.62 C, 6.75 H, 3.74 F, 8.28 N, found 68.56% C, 6.76% H, 8.12% N.

Synthesis of (S)-1-ethyl-6-fluoro-7-(4-(2-(4-isobutylphenyl)propanoyl)piperazin-1-yl)-4-oxo-N-(3-(triethoxysilyl)propyl)-1,4-dihydroquinoline-3-carboxamide, ***2c***

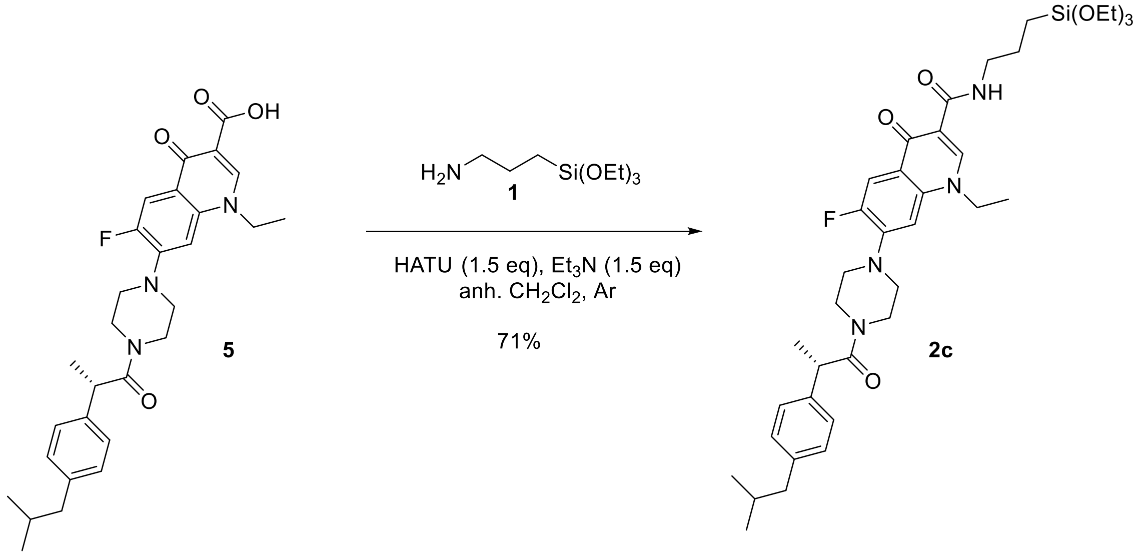

Carboxylic acid **5** (0.35 g, 0.690 mmol, 1 equiv.), Et_3N (0.10 mL, 1.00 mmol, 1.4 equiv.) and HATU (0.3 g, 0.789 mol, 1.14 equiv.) were dissolved in CH_2Cl_2 (10 mL), the mixture was stirred for 30 minutes under argon atmosphere. Then, 3-aminopropyltriethoxy silane (0.16 g, 0.723 mmol, 1.04 equiv.) was added to the solution and the solution was refluxed for 48h. The reaction was monitored by TLC. The reaction mixture was quenched with water, extracted with ethyl acetate, dried over sodium sulfate and concentrated under reduced pressure. The obtained residue was purified by silica gel column chromatography by using AcOEt : EtOH (95 : 5) as eluent to afford compound **2c** in 71 % yield (0.35 g, 0.493 mmol).

^{1}H NMR (400 MHz, $CDCl_3$) δ (ppm): 9.98 (t, *J* = 5.8 Hz, 1H), 8.65 (s, 1H), 7.97 (d, *J* = 13.1 Hz, 1H), 7.12 (d, *J* = 7.8 Hz, 2H), 7.05 (d, *J* = 7.8 Hz, 2H), 6.63 (d, *J* = 6.7 Hz, 1H), 4.18 (q, *J* = 7.3 Hz, 2H), 4.01 (m, 1H), 3.85 (m 1H), 3.77 (q, *J* = 7.0 Hz, 6H), 3.65 (m, 1H), 3.54 (m, 2H), 3.40 (app q, *J* = 6.8, 2H), 3.19 (m, 1H), 3.07 (m, 2H), 2.56 (m, 1H), 2.38 (d, *J* = 7.2 Hz, 2H), 1.85 – 1.64 (m, 3H), 1.51 – 1.37 (m, 6H), 1.17 (t, *J* =7.0 Hz, 9H), 0.81 (m, 6H), 0.67 (t, *J* = 8.2 Hz, 2H). **^{13}C NMR** (101 MHz, $CDCl_3$) δ (ppm): 175.3, 172.4, 164.8, 153.0 (d, *J* = 249.5 Hz), 146.6, 144.5 (d, *J* = 11.1 Hz), 140.4, 139.0, 136.4, 129.8, 126.92, 122.9 (d, *J* = 7.1 Hz), 113. 0 (d, J = 23.2 Hz), 111.8, 103.9 (d, *J* = 3.0 Hz), 58.4, 50.0, 49.0, 45.3, 45.0, 43.0, 42.0, 41.6, 30.2, 23.3, 22.34, 22.30, 20.7, 18.3, 14.4, 8.0. **IR** (ATR) ν (cm^{-1}): 3240, 2970, 2925, 1650, 1541, 1487, 1229, 1074, 775. **HRMS** (ESI) m/z $[M+H]^+$ calcd for $C_{38}H_{56}FN_4O_6Si$: 711.3948 found: 711.3946.

3. NMR and IR spectra

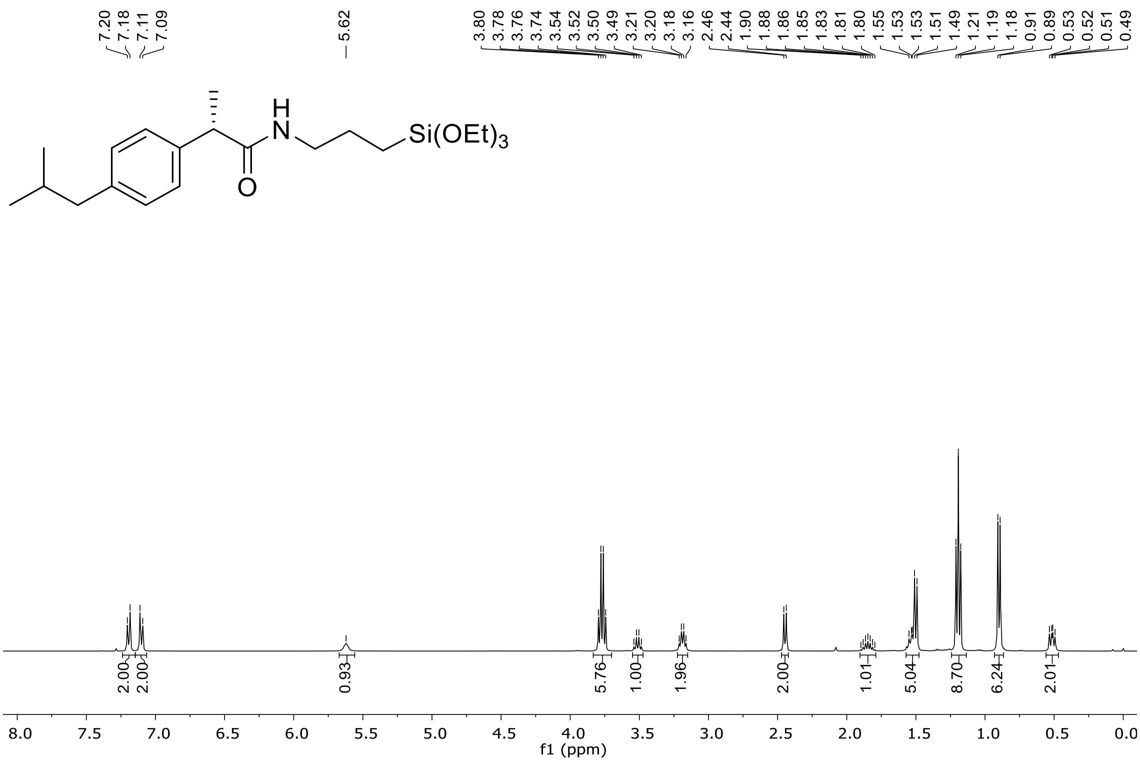

^{1}H NMR (400 MHz, $CDCl_3$) of compound **2a**

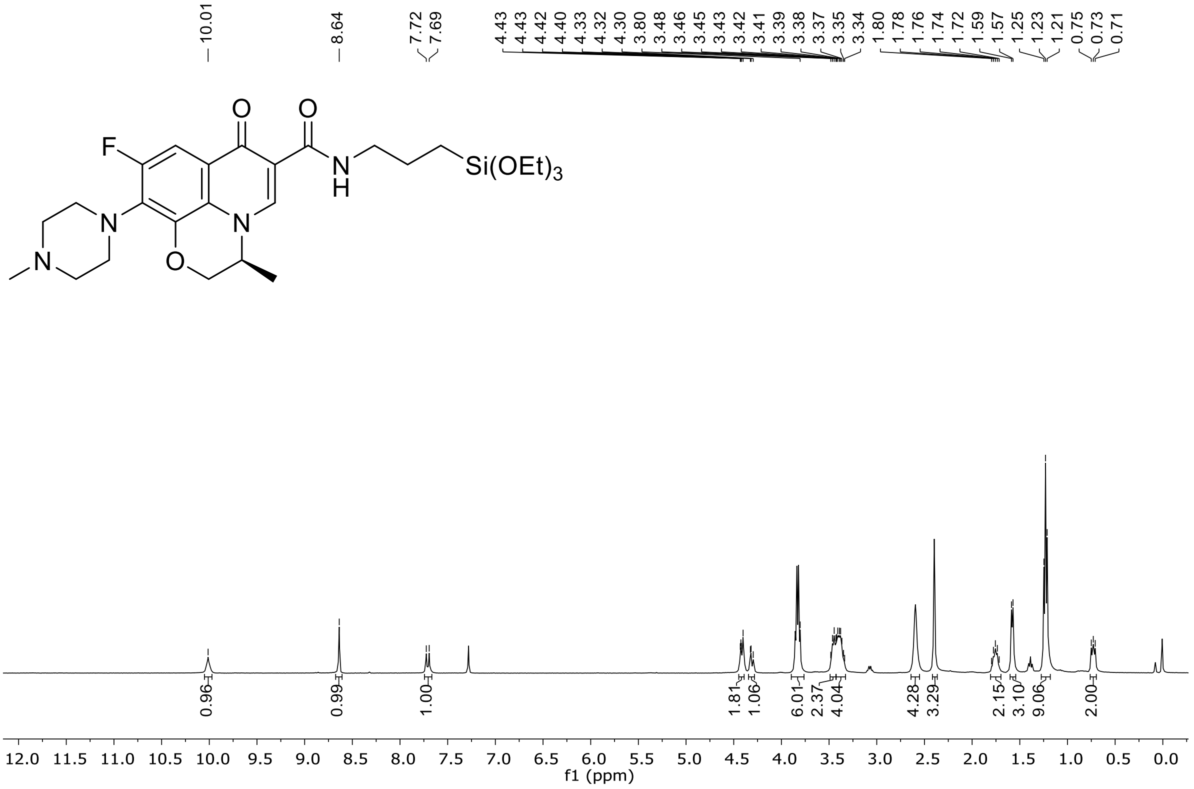

^{1}H NMR (400 MHz, $CDCl_3$) of compound **2b**

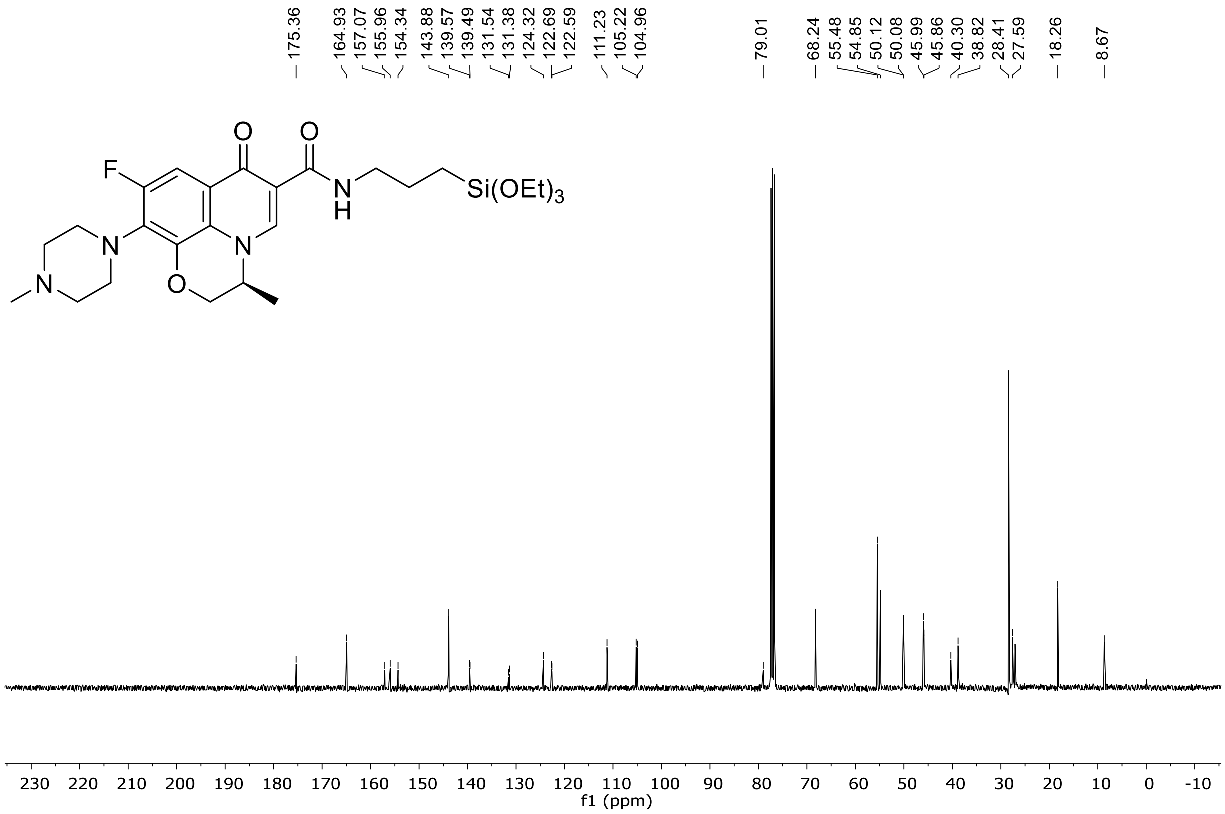

^{13}C NMR (101 MHz, $CDCl_3$) of compound **2b**

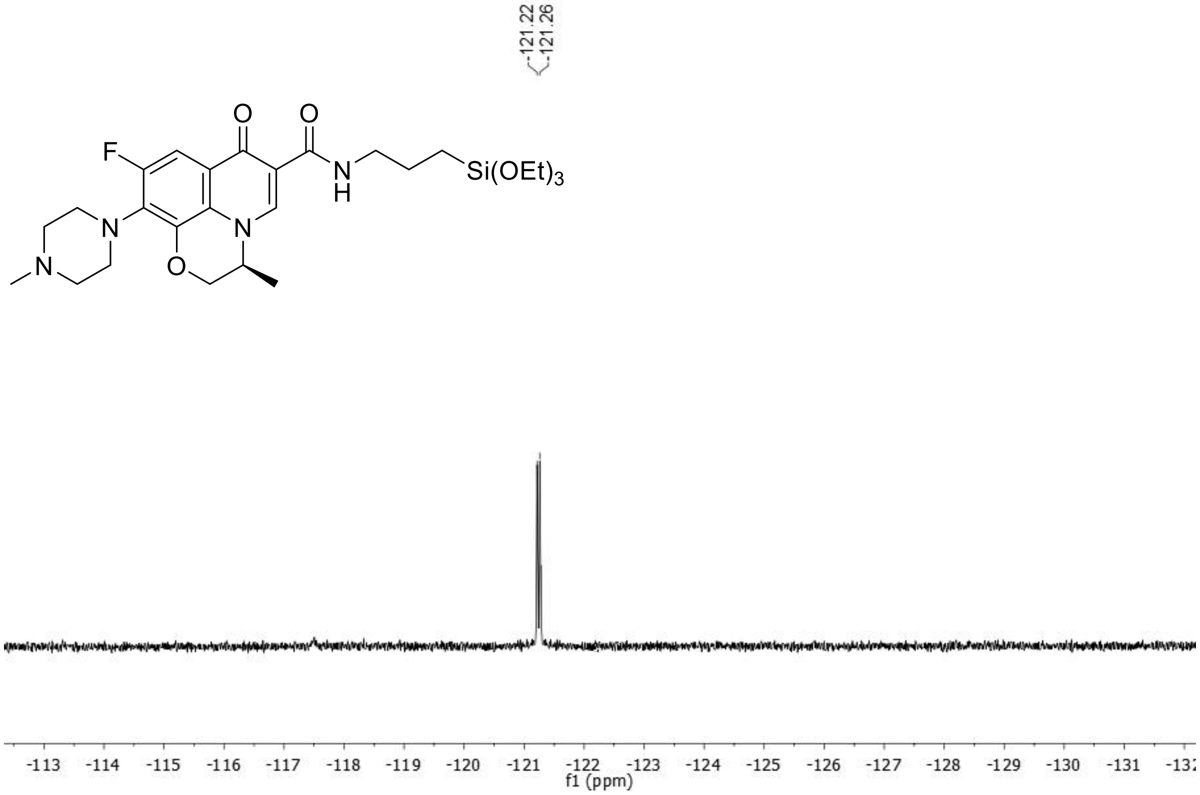

^{19}F NMR (376 MHz, $CDCl_3$) of compound **2b**

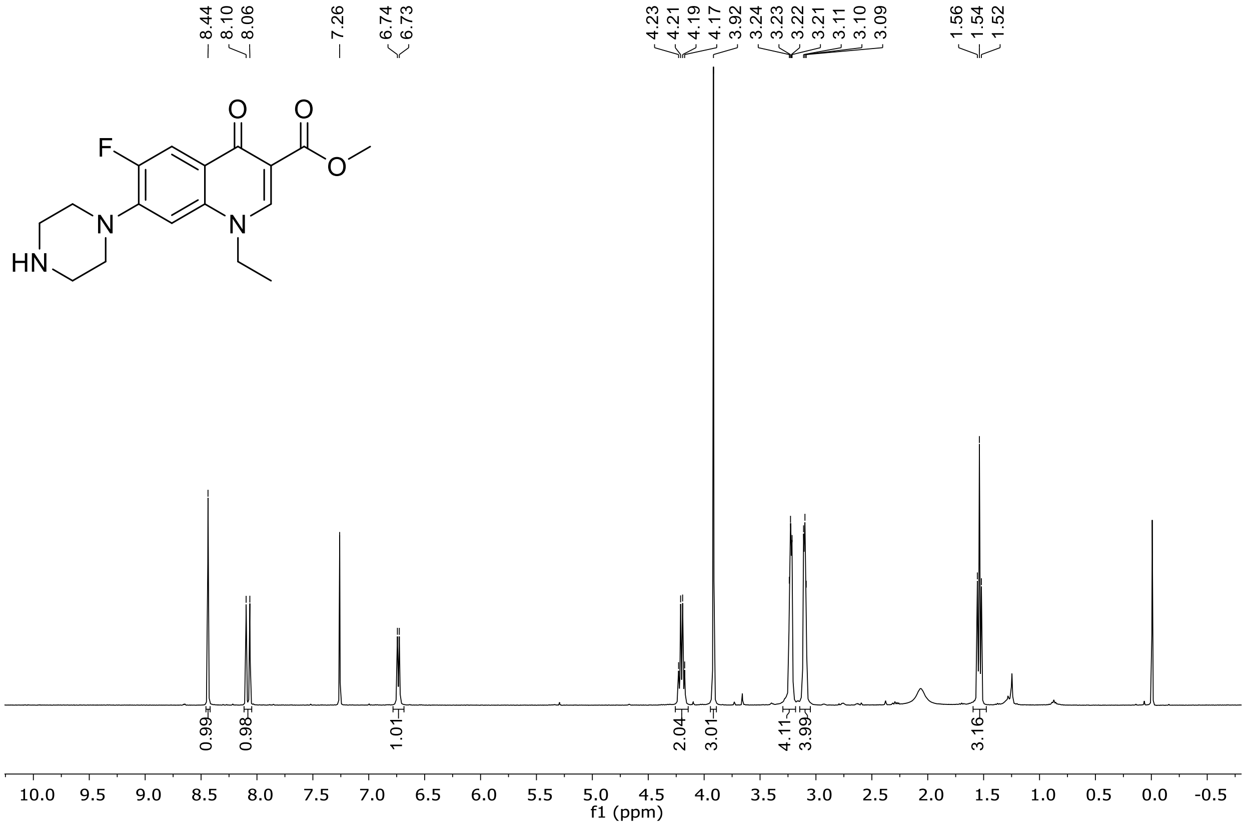

^1H NMR (400 MHz, $CDCl_3$) of compound **3**

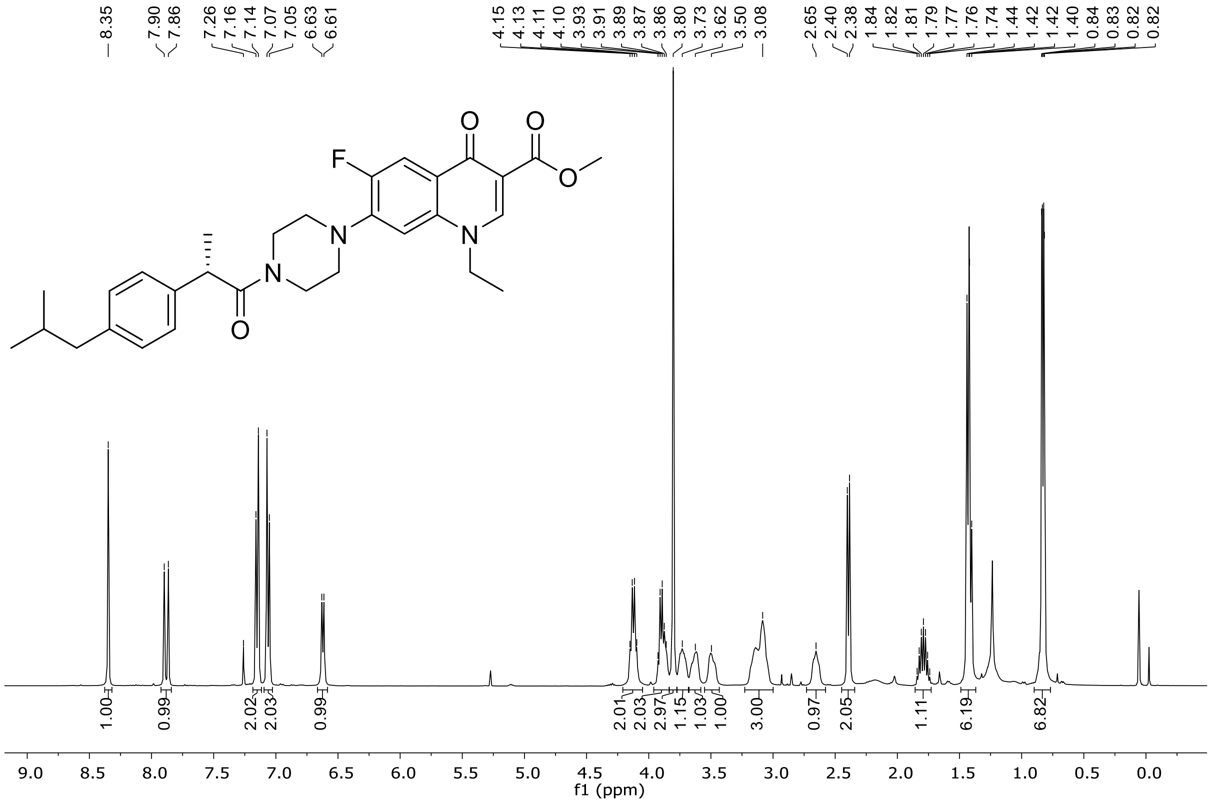

^1H NMR (400 MHz, $CDCl_3$) of compound **4**

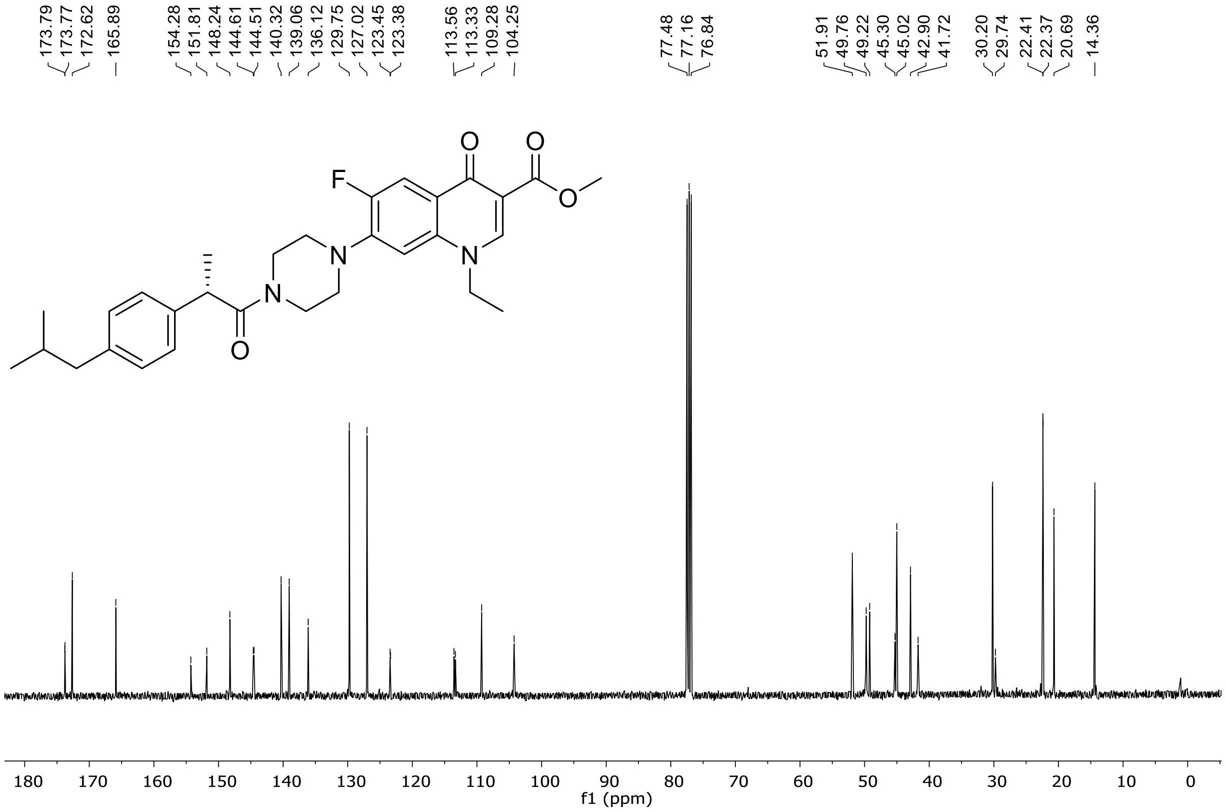

^{13}C NMR (101 MHz, $CDCl_3$) of compound **4**

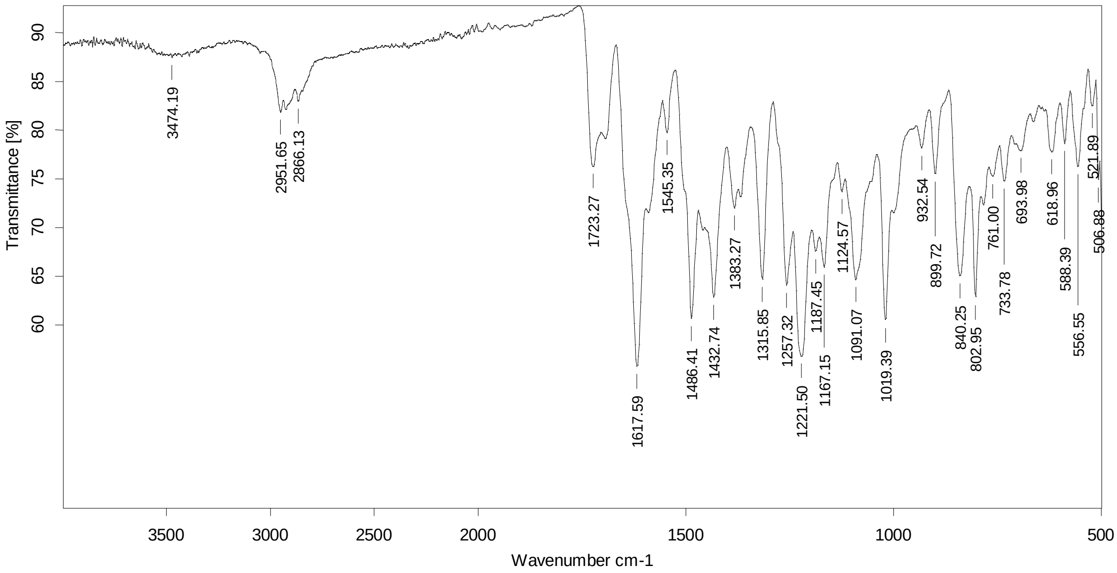

IR (ATR) of compound **4**

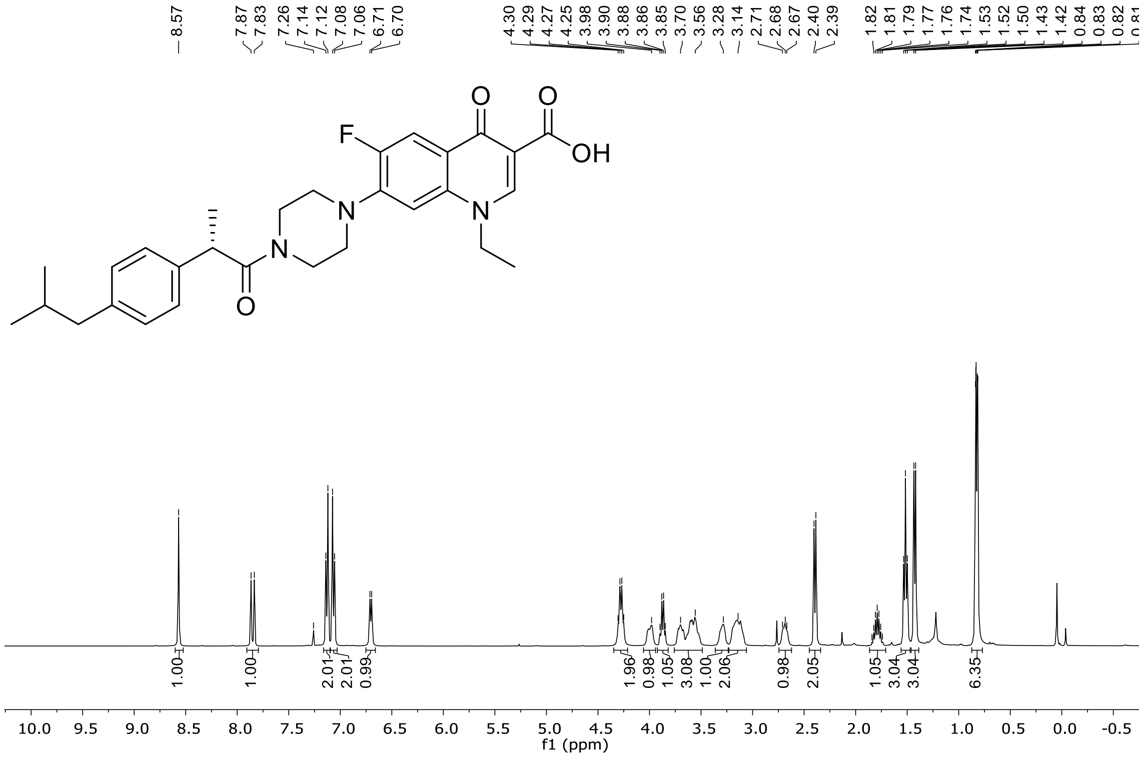

^{1}H NMR (400 MHz, $CDCl_3$) of compound **5**

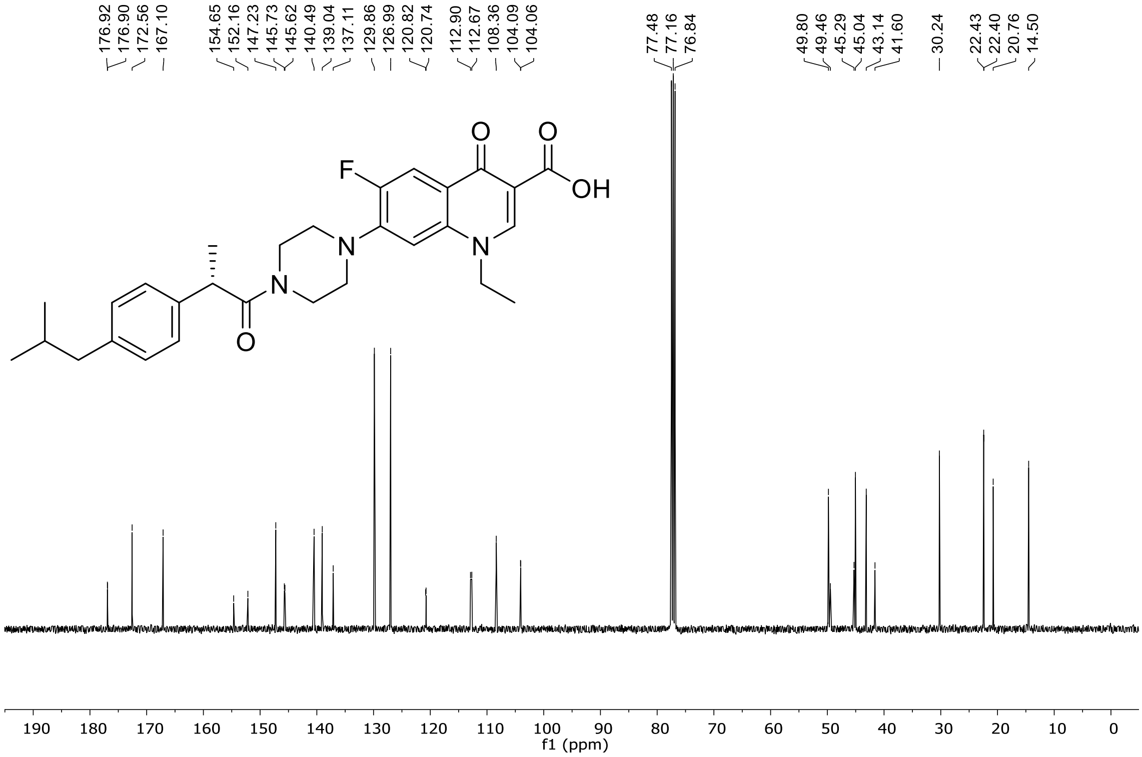

^{13}C NMR (101 MHz, $CDCl_3$) of compound **5**

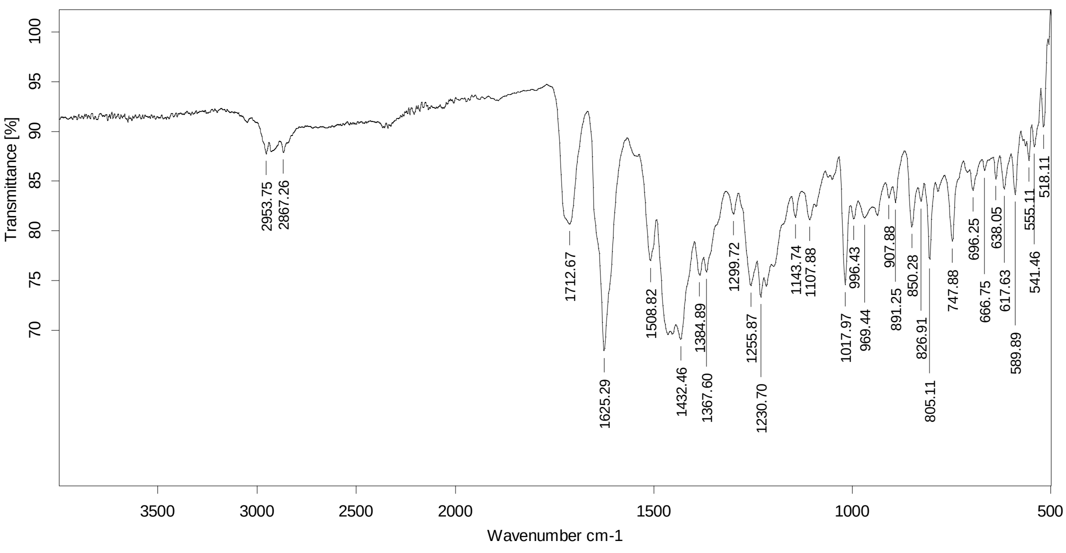

IR (ATR) of compound **5**

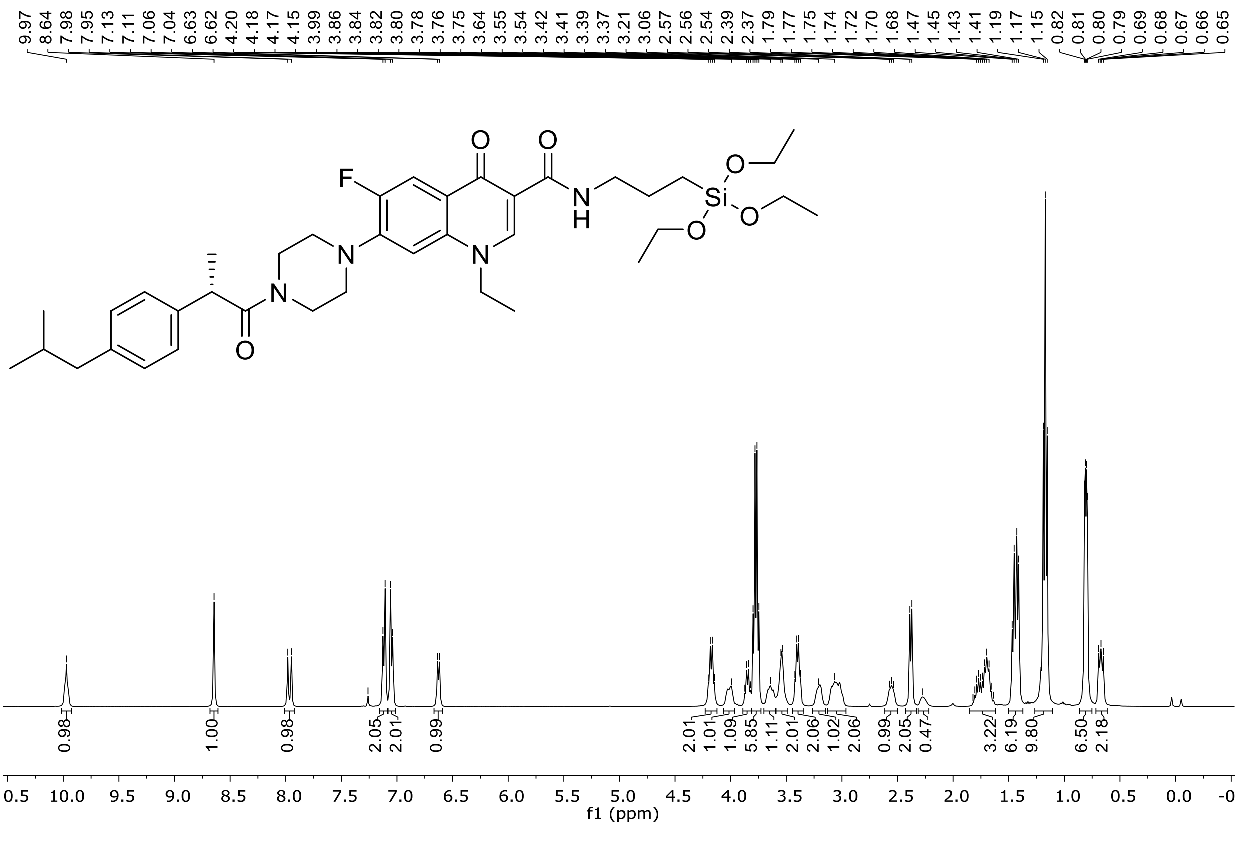

^{1}H NMR (400 MHz, $CDCl_3$) of compound **2c**

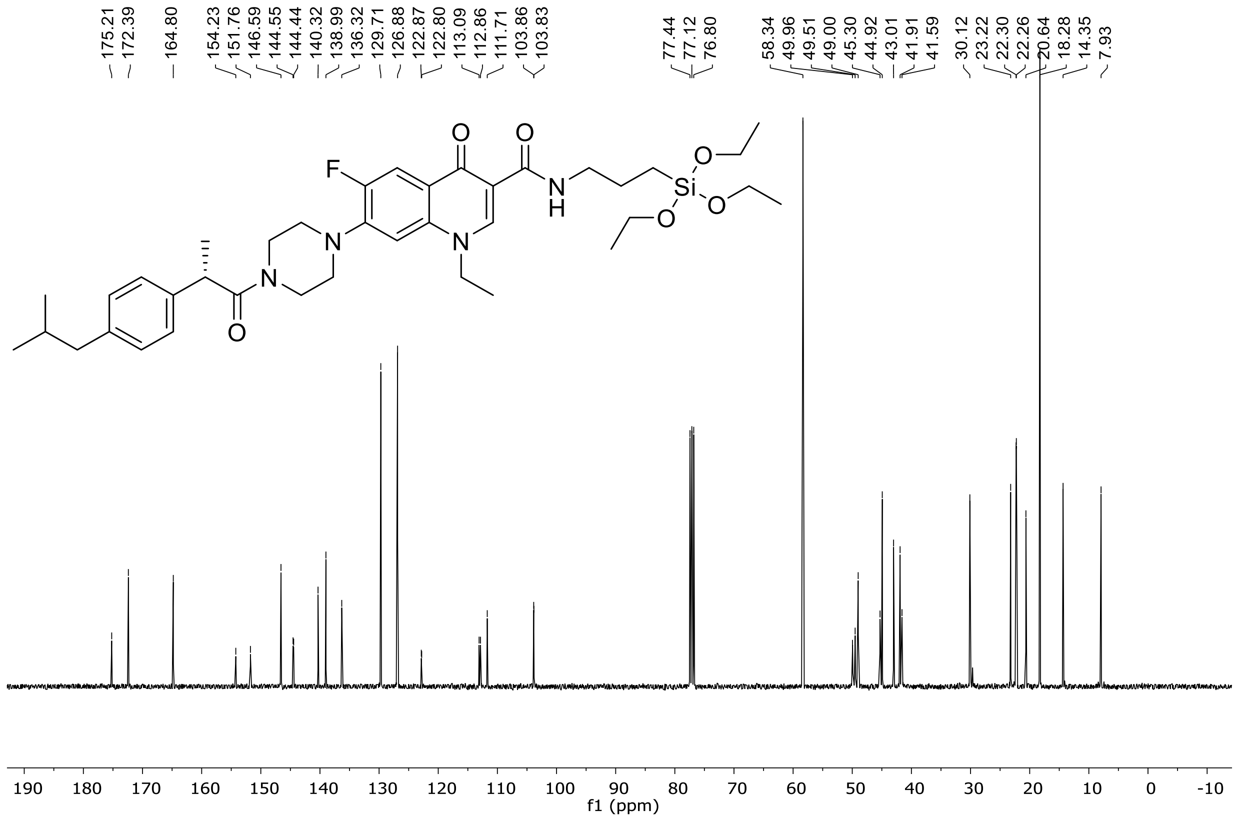

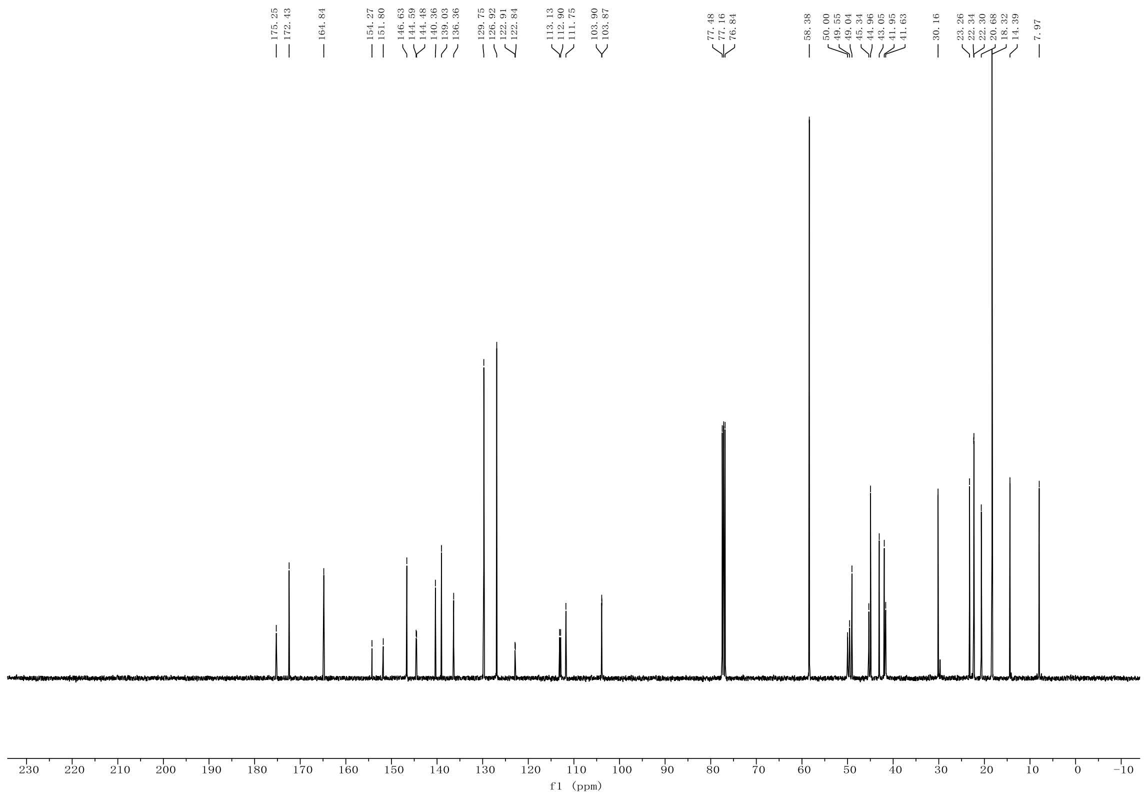

^{13}C NMR (101 MHz, $CDCl_3$) of compound **2c**

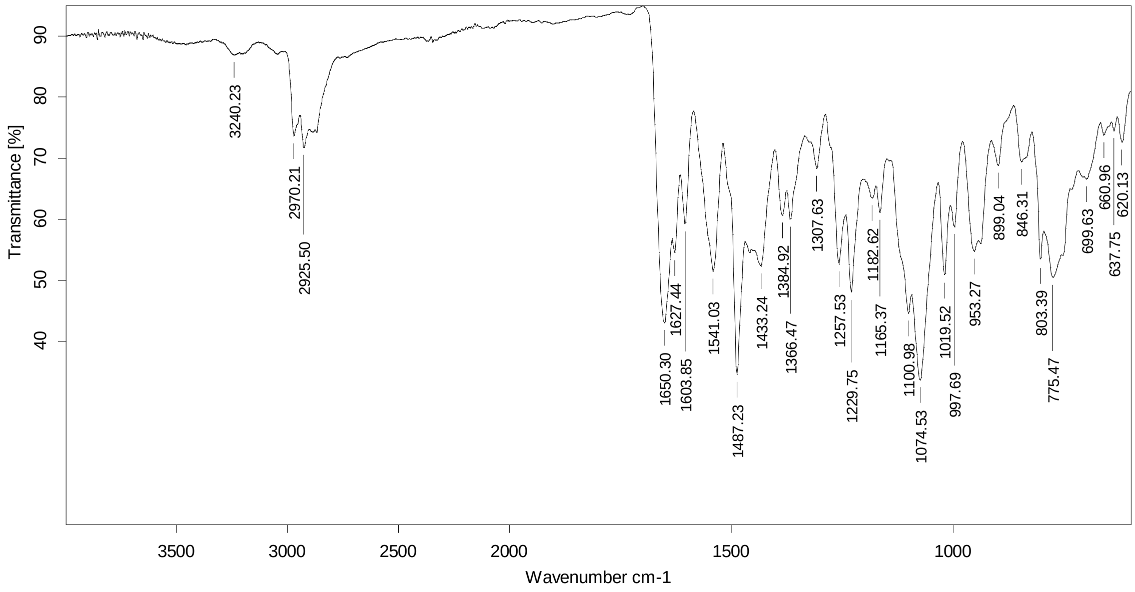

IR (ATR) of compound **2c**

4. Preparation and characterization of functionalized silica nanoparticles and fabrics

Preparation of SiO_2@Ibu+Leflox (20:0.5:0.5)

TEOS (2.08 g, 10.0 mmol), the silylated anti-inflammatory ibuprofen **2a** (0.25 mmol) and silylated antibiotic levofloxacin **2b** (0.25 mmol) were dissolved in absolute EtOH (25 mL). Then, an ammonium hydroxide-ethanol solution (6 mL of 28% $NH_3 \cdot H_2O$ in 25 mL EtOH) was added. The mixture was magnetically stirred (1400 rpm) at room temperature for 12 hours. Then the functionalized nanoparticles were collected by centrifugation (13500 rpm for 10 minutes) and washed with ethanol until neutral pH was reached. Then, the obtained solid was washed successively with Mili-Q water and 96% ethanol, and it was dried under vacuum for several hours.

Preparation of SiO_2@Ibu+Leflox (20:0.75:0.25)

TEOS (3.53 g, 16.97 mmol), the silylated anti-inflammatory ibuprofen **2a** (0.63 mmol) and silylated antibiotic levofloxacin **2b** (0.21 mmol) were dissolved in absolute EtOH (42 mL). Then, an ammonium hydroxide-ethanol solution (10 mL of 28% $NH_3 \cdot H_2O$ in 42 mL EtOH) was added. The mixture was magnetically stirred (1400 rpm) at room temperature for 12 hours. Then the functionalized nanoparticles were collected by centrifugation (13500 rpm for 10 minutes) and washed with ethanol until neutral pH was reached. Then, the obtained solid was washed successively with Mili-Q water and 96% ethanol, and it was dried under vacuum for several hours.

SiO_2@Ibu+Leflox (20:0.5:0.5): EA: 9.26% C, 2.02% H, 1.61% N (**2a** 0.23 mmol/g material **2b** 0.23 mmol/g material). IR ν (ATR): 3274.8, 1051.1, 948.5, 800.1. DLS: 2733 nm, Zeta-potential: ζ = 5.7 mV.

SiO_2@Ibu+Leflox (20:0.75: 0.25): EA: 4.20% C, 1.83% H, 0.25% N (**2a** 0.075 mmol/g material **2b** 0.025 mmol/g material). IR ν (ATR): 3302.3, 1054.3, 947.0, 796.1, 545.3. DLS: 426 nm, Zeta-potential: ζ = -41.0 mV.

General procedure for the preparation of SiO_2@Norflox-Ibu

TEOS (2.08 g, 10.0 mmol) and the silylated derivative **2c** (0.5, 0.33 and 0.25 mmol) were dissolved in absolute EtOH (25 mL). Then, an ammonium hydroxide-ethanol solution was added (6 mL of 28% NH_3 H_2O in 25 mL EtOH). The mixture was magnetically stirred (1400 rpm) at room temperature for 12 hours. The functionalized nanoparticles were collected by centrifugation

(13500 rpm for 10 minutes) and washed with ethanol until neutral pH was reached. Then, the obtained white solid was washed successively with Mili-Q water and 96% ethanol, and the nanomaterials were dried under high vacuum. We obtained **SiO_2@Norflox-Ibu** with different ratios of TEOS and silylated drug **2c**.

SiO_2@Norflox-Ibu (20:1): EA: 1.96% C, 1.52% H, 0.86% N (0.15 mmol/g material). IR ν (ATR): 3409.3, 1631.0, 1052.4, 948.1, 794.4, 522.7. Solid state ^{13}C-CP-MAS NMR (100.6 MHz) δ (ppm): 175.6, 166.9, 140.0, 129.8, 111.4, 59.3, 45.3, 30.6, 22.1, 17.6, 14.2. ^{29}Si-CP-MAS NMR (79.5 MHz) δ (ppm): -58.35 (T^2), -66.77 (T^3), -102.34 (Q^3), -112.08 (Q^4). DLS: 836 nm, Zeta-potential: ζ = -65.5 mV.

SiO_2@Norflox-Ibu (30:1): EA: 2.52% C, 1.69% H, 0.74% N (0.13 mmol/g material). IR ν (ATR): 3283.5, 1637.8, 1058.2, 953.5, 795.9, 543.6. DLS: 658 nm, Zeta-potential: ζ = -69.9 mV.

SiO_2@Norflox-Ibu (40:1): EA: 1.61% C, 1.63% H, 0.84% N (0.15 mmol/g material). IR ν (ATR): 3374.0, 1630.1, 1053.6, 949.4, 792.7, 565.7. ^{29}Si-CP-MAS NMR (79.5 MHz) δ (ppm): -56.53 (T^2), -65.38 (T^3), -92.36 (Q^2), -101.83 (Q^3), -112.39 (Q^4). DLS: 486 nm, Zeta-potential: ζ = -72.3 mV.

SiO_2@Ibu+Leflox (20:0.5:0.5)

DLS

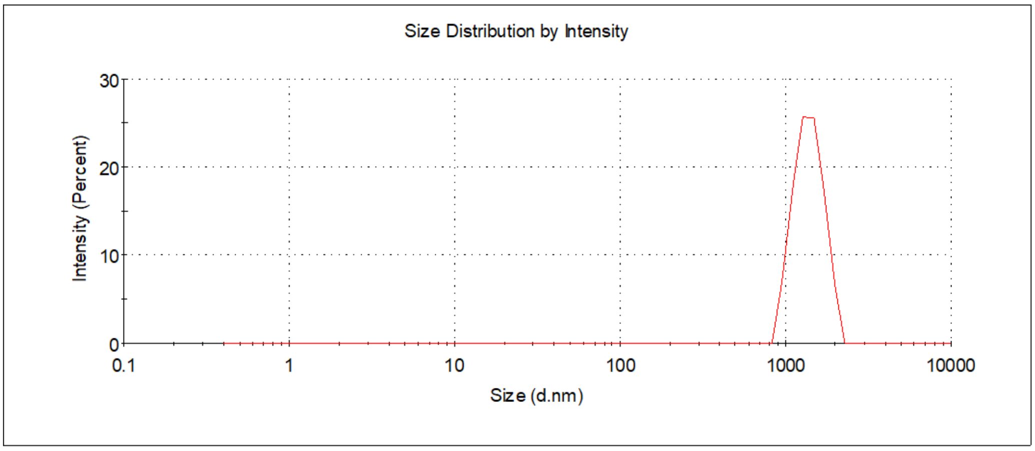

Zeta-potential

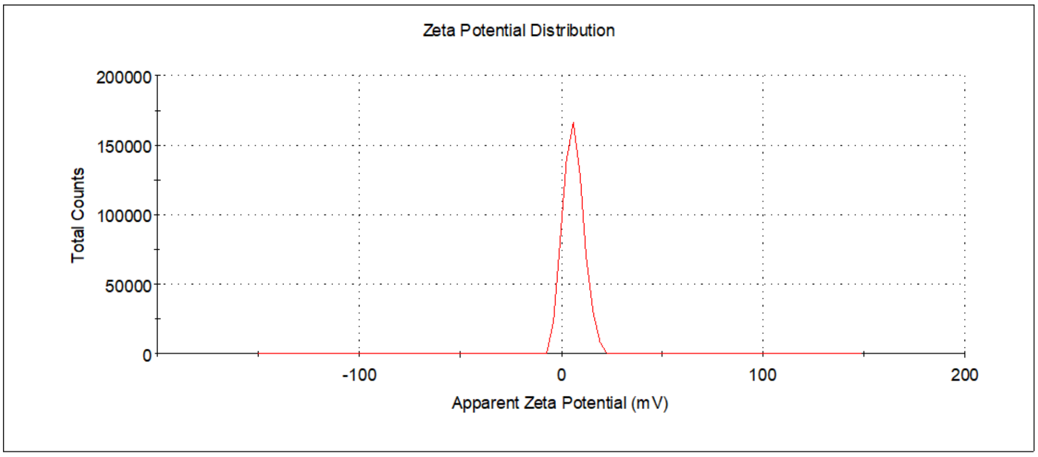

TEM

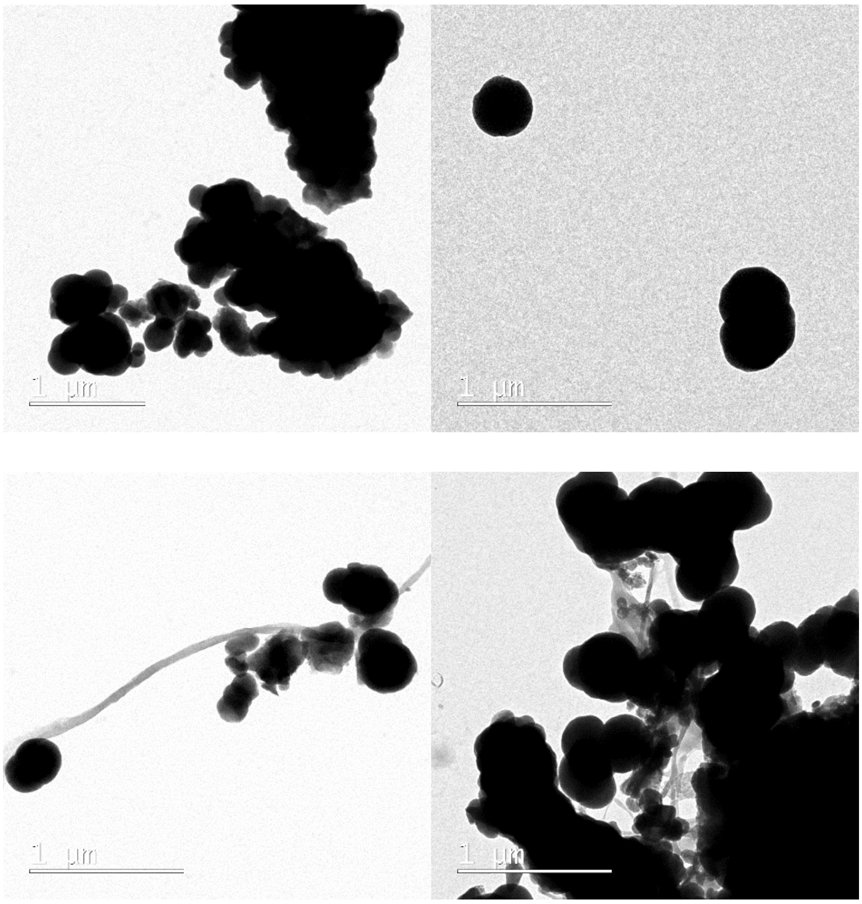

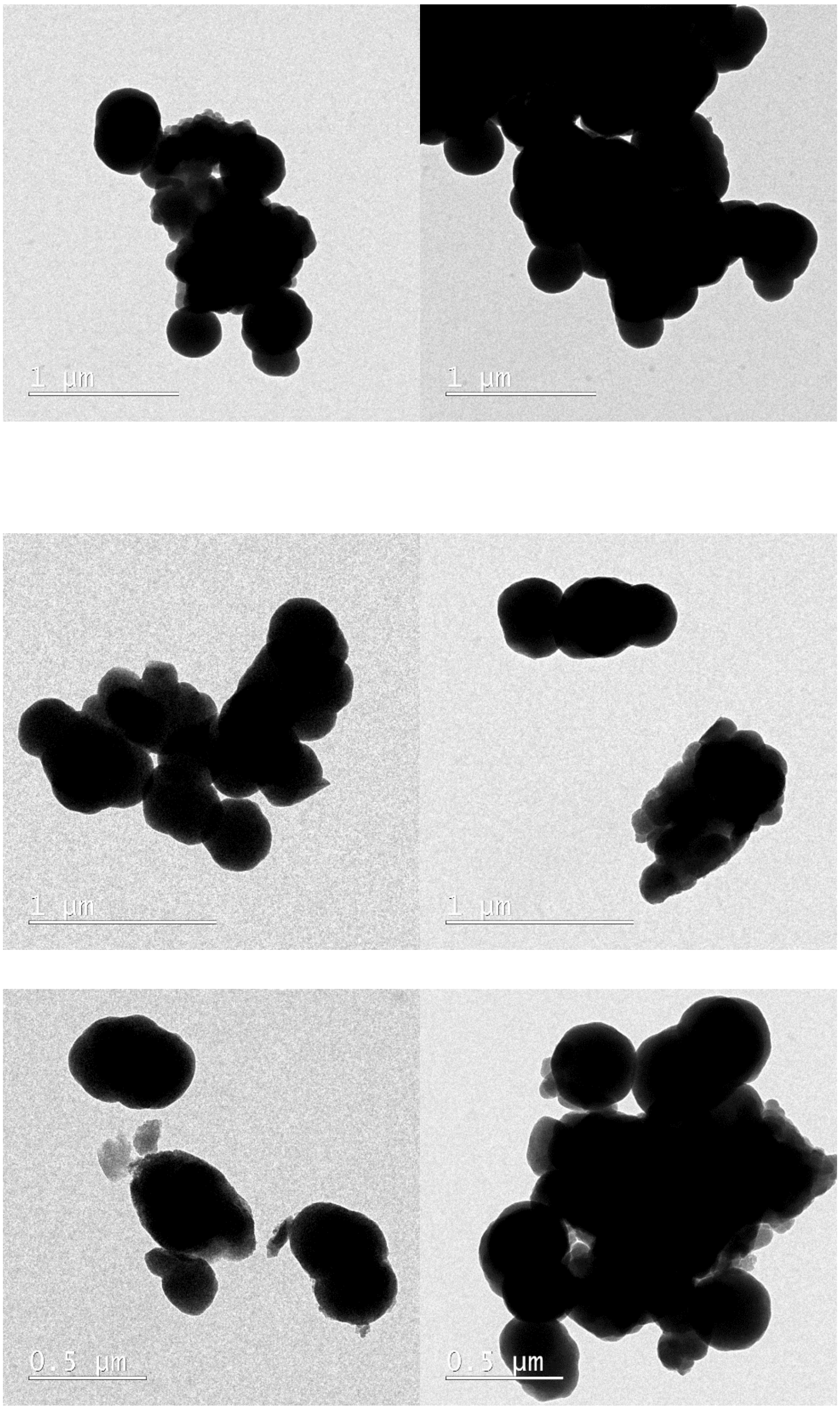
1 μm
1 μm
1 μm
1 μm
0.5 μm
0.5 μm

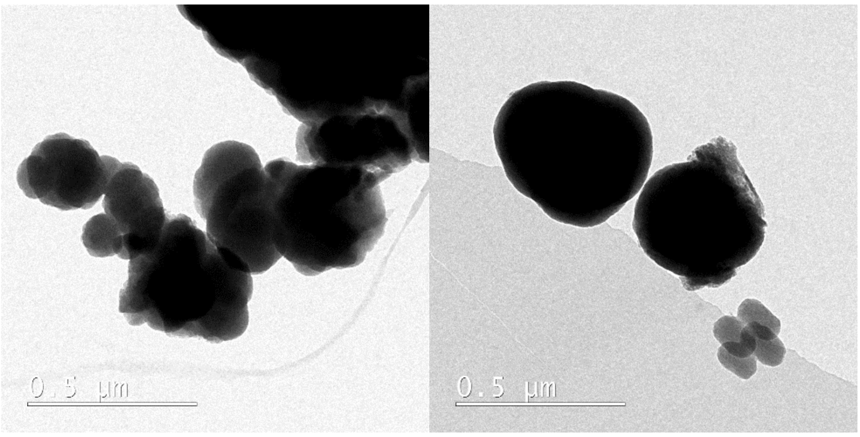
0.5 µm
0.5 µm

IR (ATR)

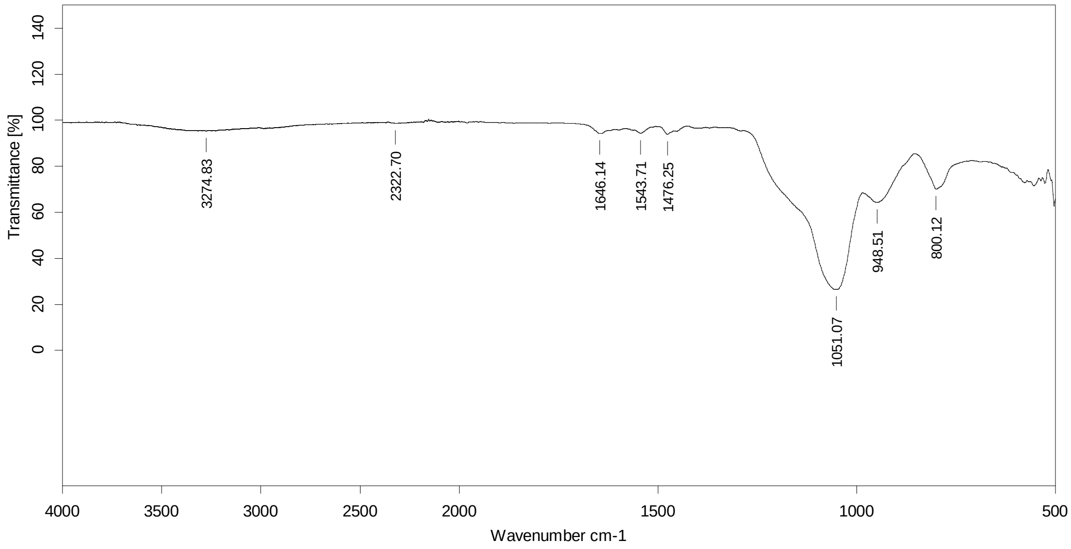
Transmittance [%]
140
120
100
80
60
40
20
0
3274.83
2322.70
1646.14
1543.71
1476.25
1051.07
948.51
800.12
4000
3500
3000
2500
2000
1500
1000
500
Wavenumber cm-1

SiO_2@Ibu+Leflox (20:0.75: 0.25)

DLS

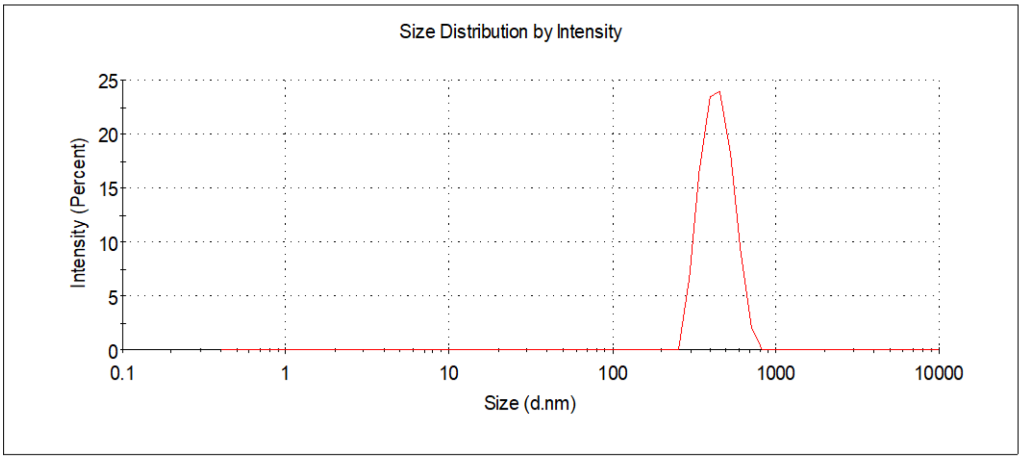

Zeta-potential

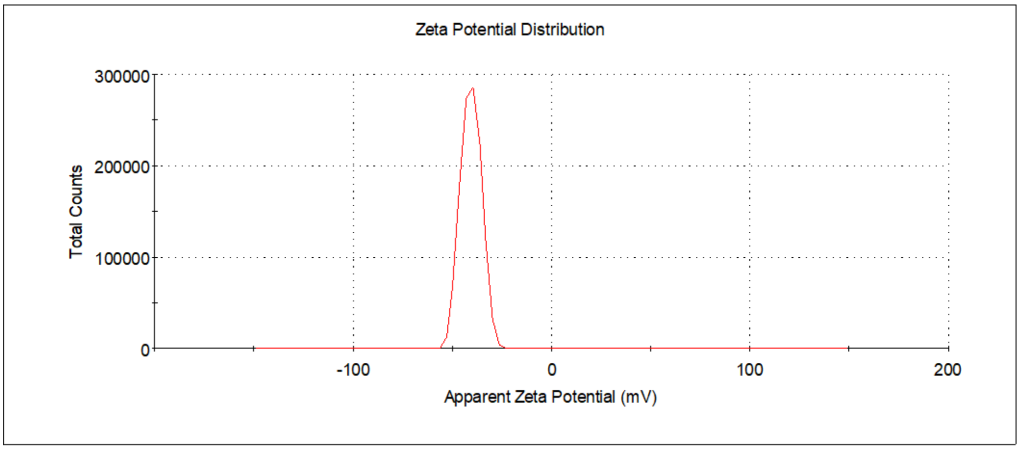

TEM

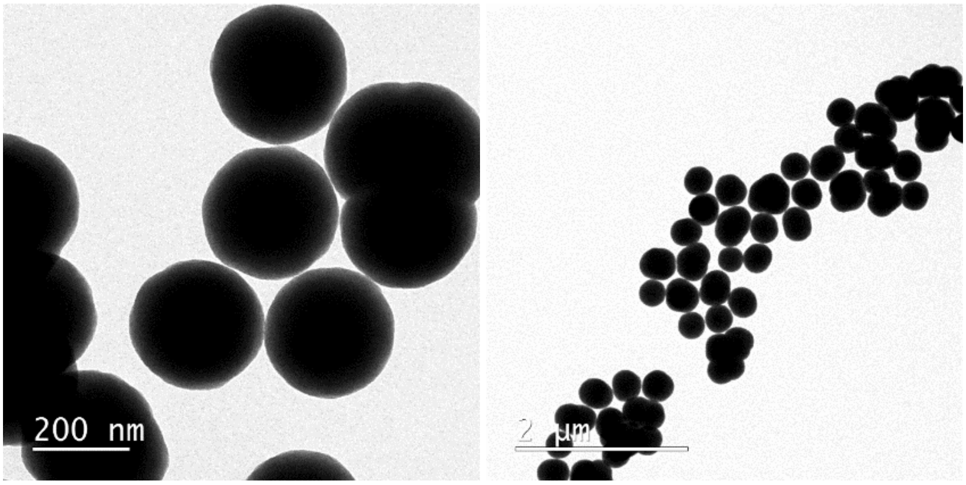
200 nm
2 µm

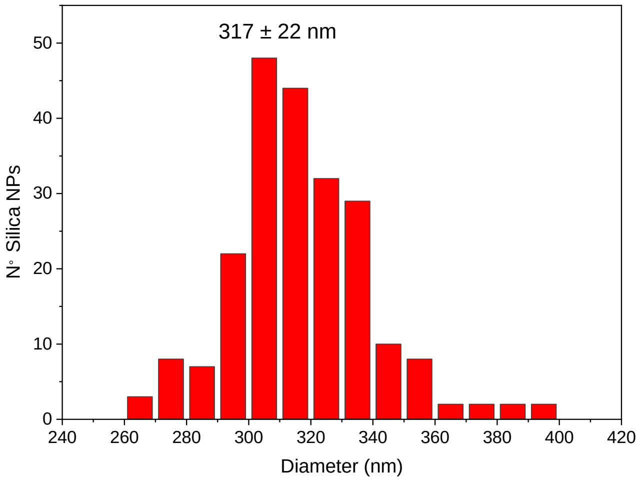
317 ± 22 nm
N° Silica NPs
Diameter (nm)
0
10
20
30
40
50
240
260
280
300
320
340
360
380
400
420

ATR

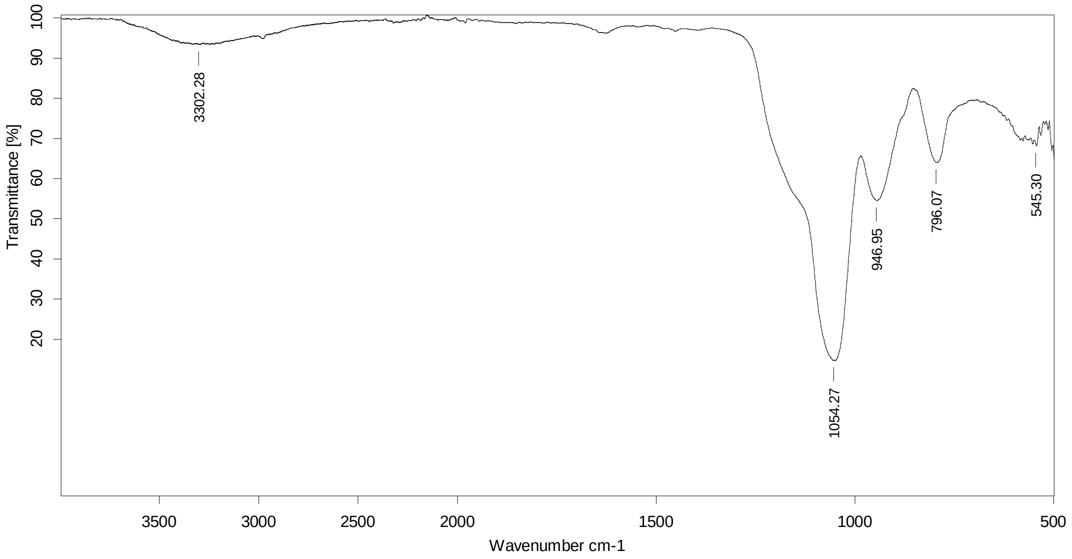
Transmittance [%]
Wavenumber cm-1
3302.28
1054.27
946.95
796.07
545.30
20
30
40
50
60
70
80
90
100
3500
3000
2500
2000
1500
1000
500

SiO_2@Norflox-Ibu (TEOS: 2c (20:1))

DLS

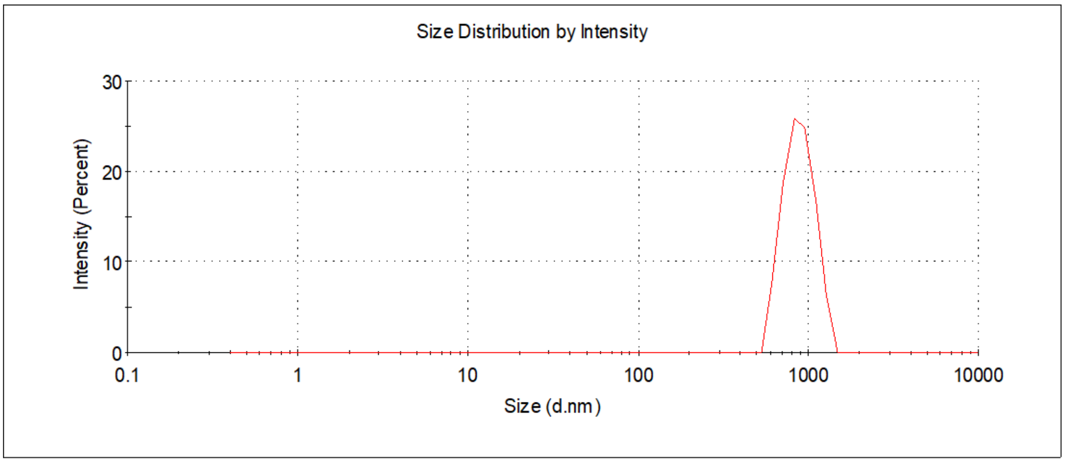

Zeta-potential

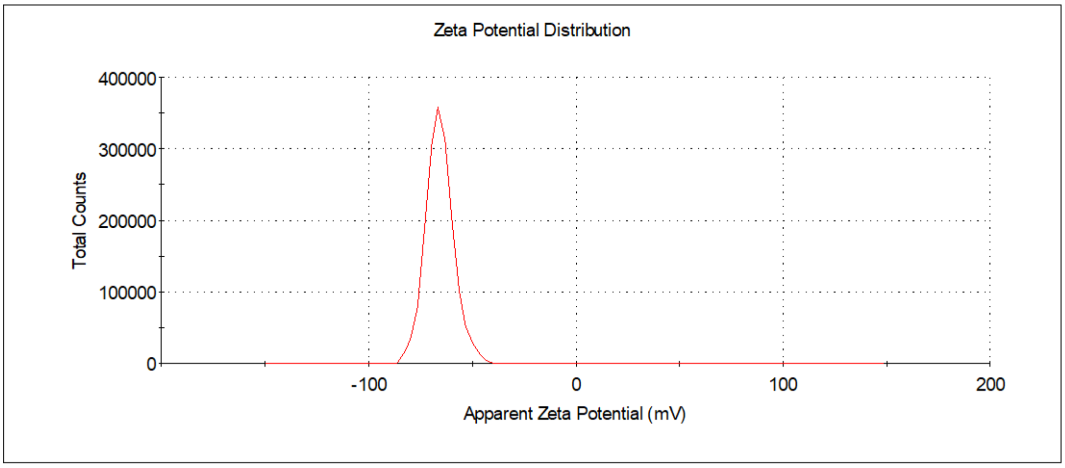

TEM

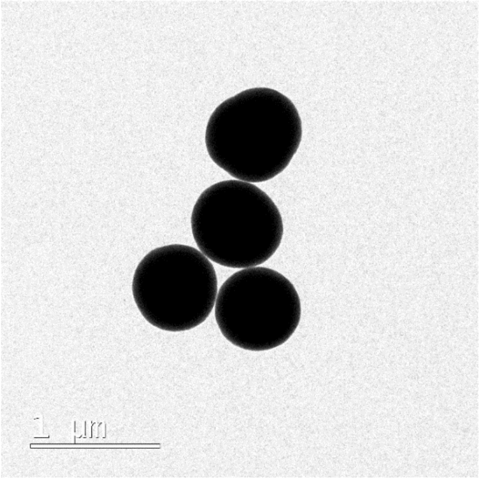

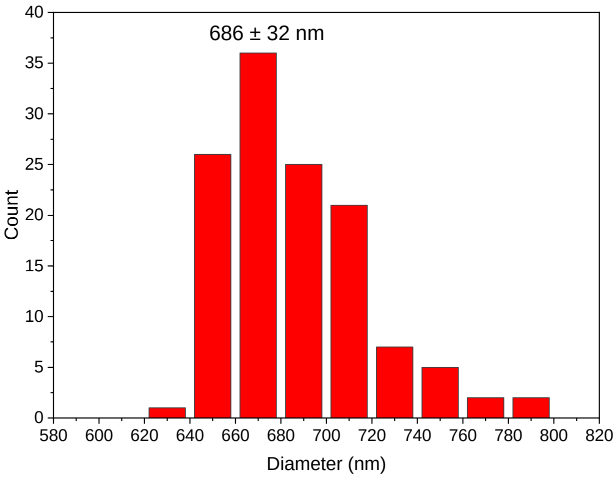

Solid state ^{13}C NMR and ^{29}Si NMR

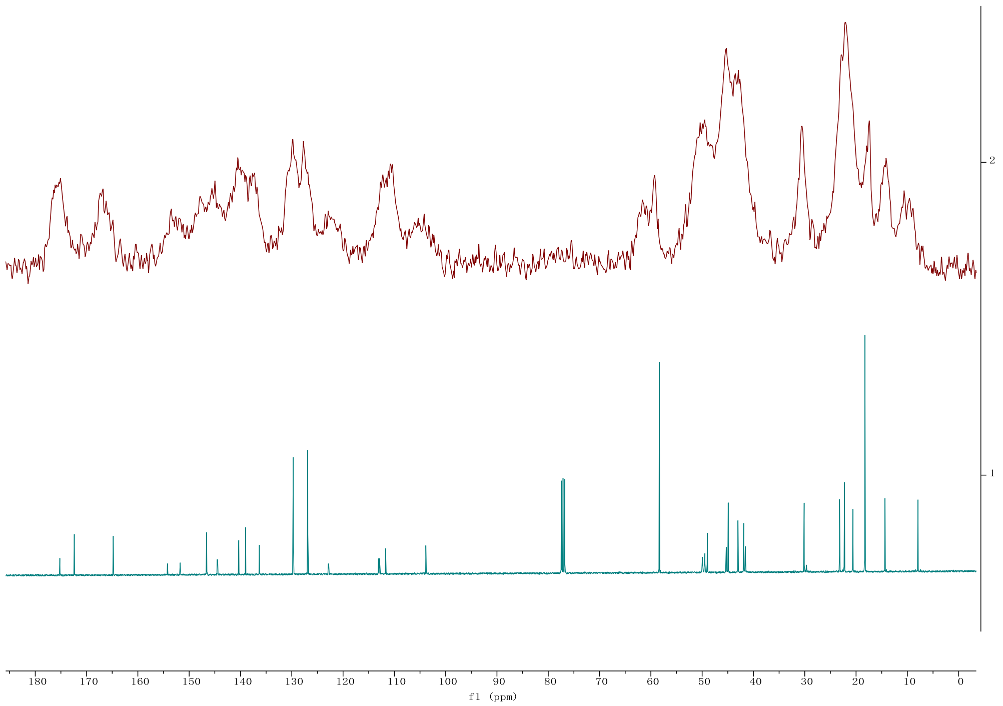

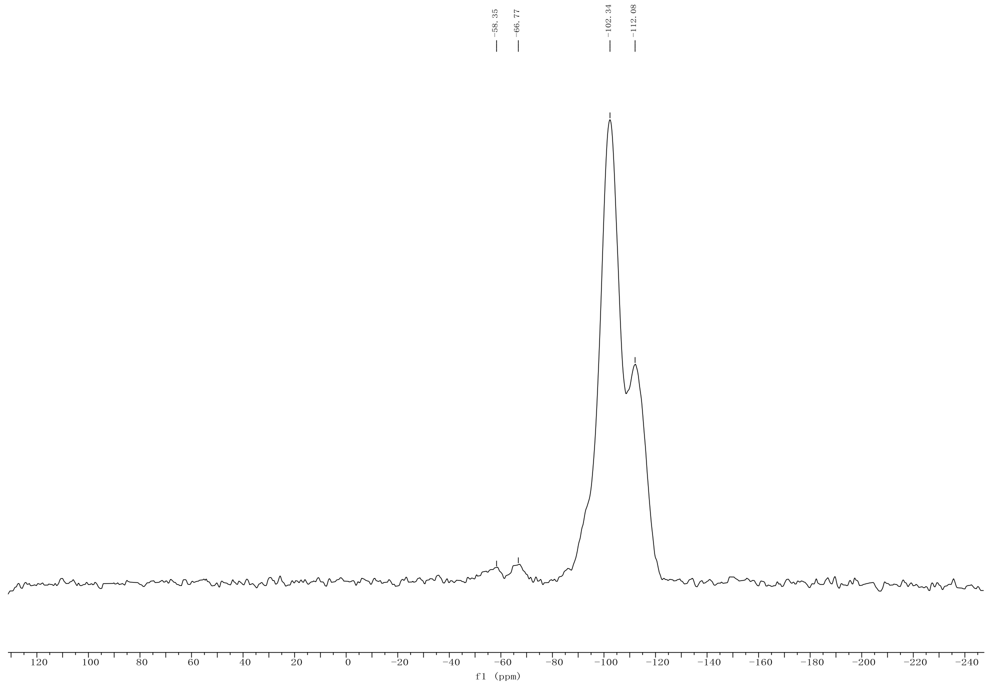

IR (ATR)

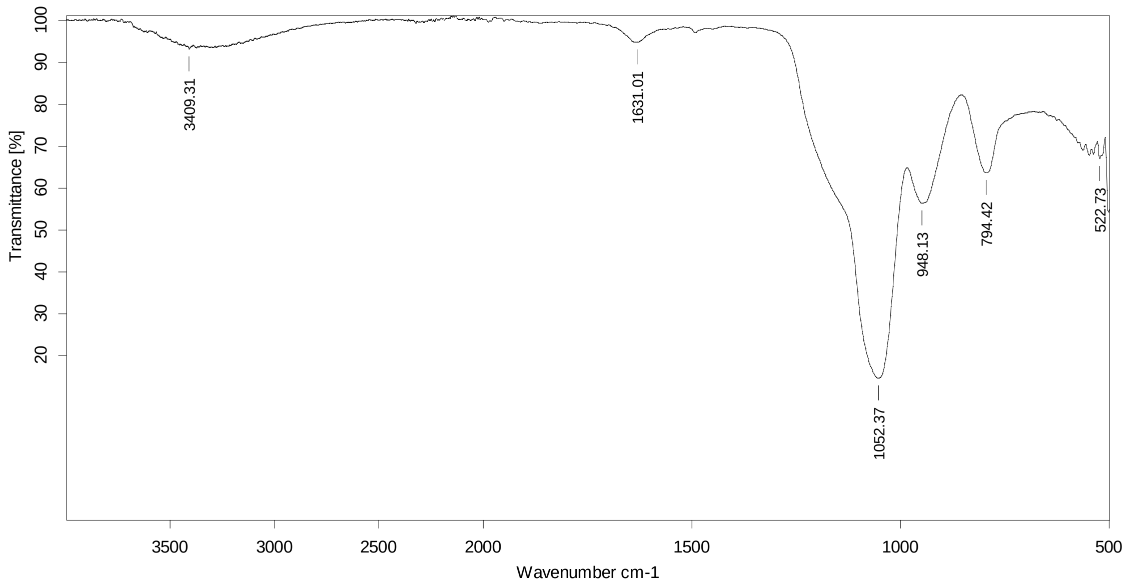

SiO_2@Norflox-Ibu (TEOS: 2c (30:1))

DLS

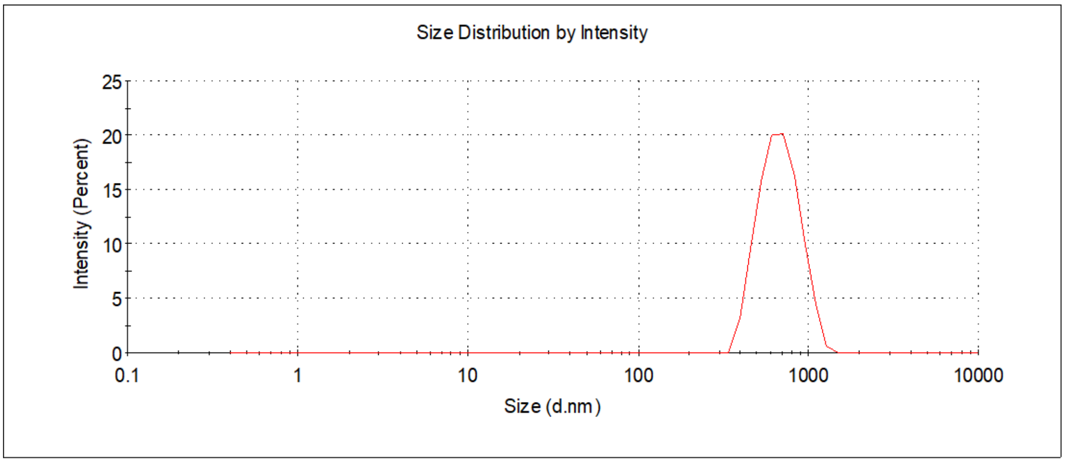

Zeta-potential

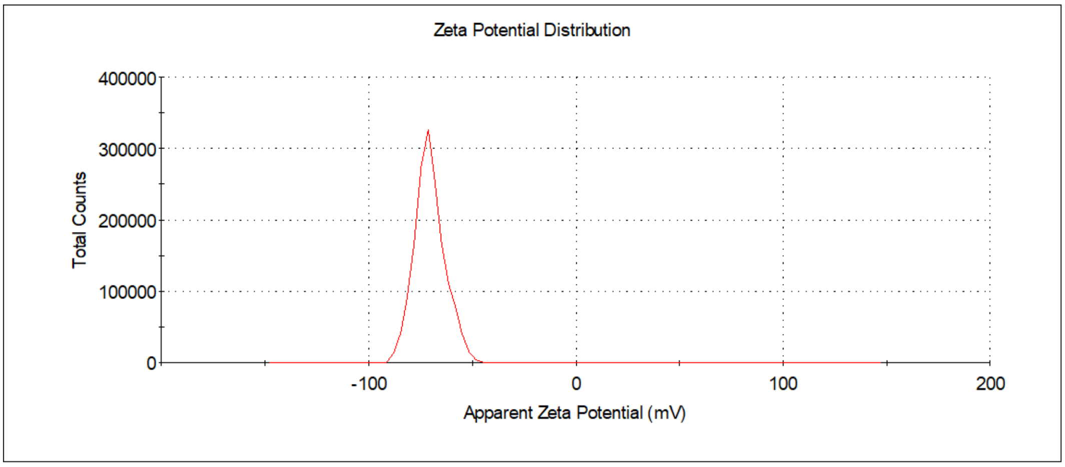

TEM

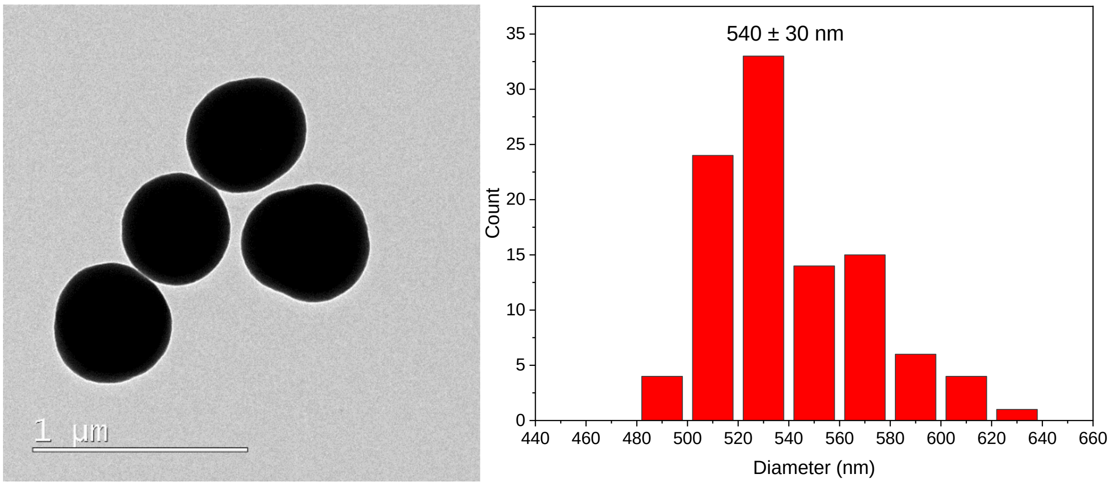

IR (ATR)

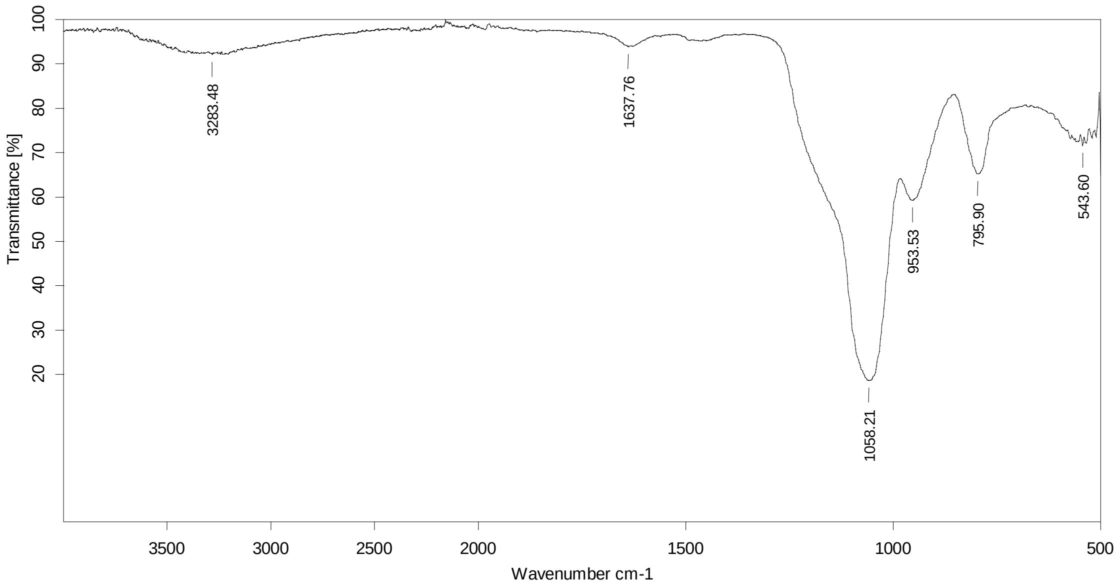

SiO_2@Norflox-Ibu (TEOS: 2c (40:1))

DLS

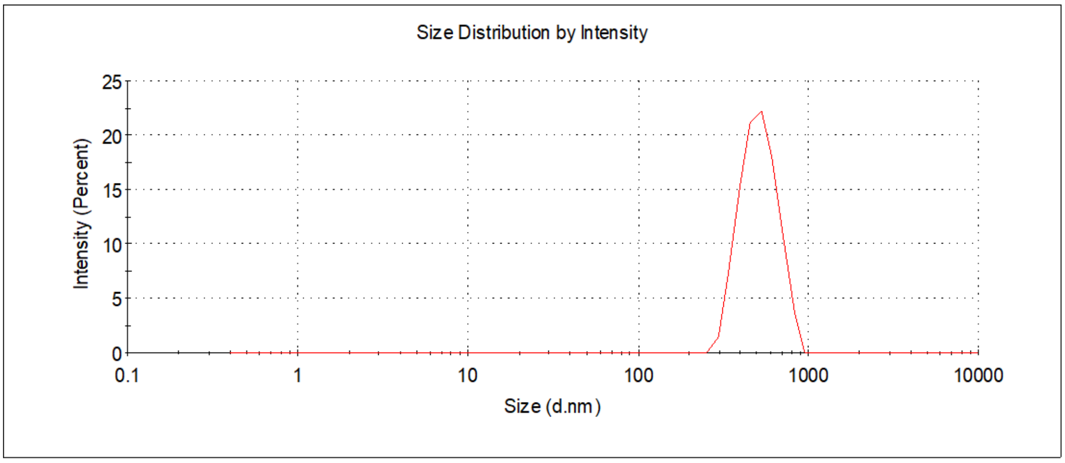

Zeta-potential

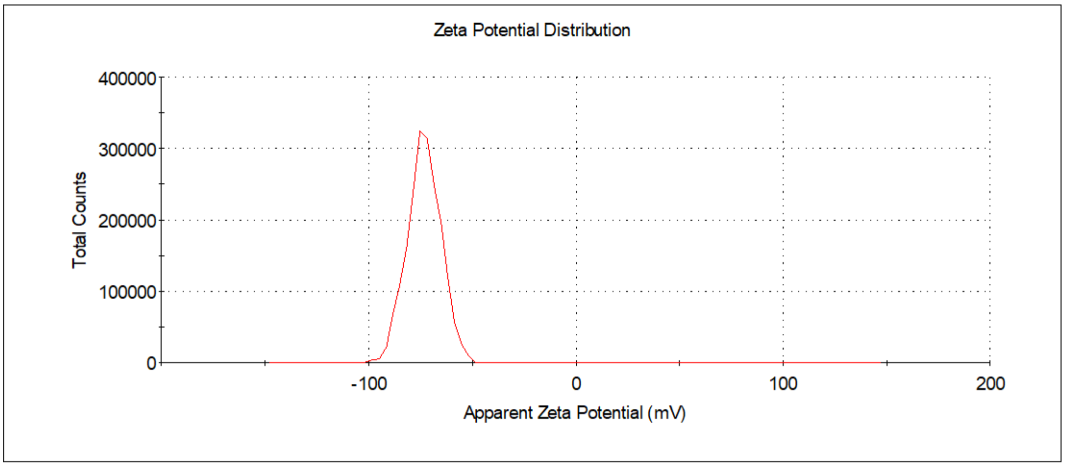

TEM

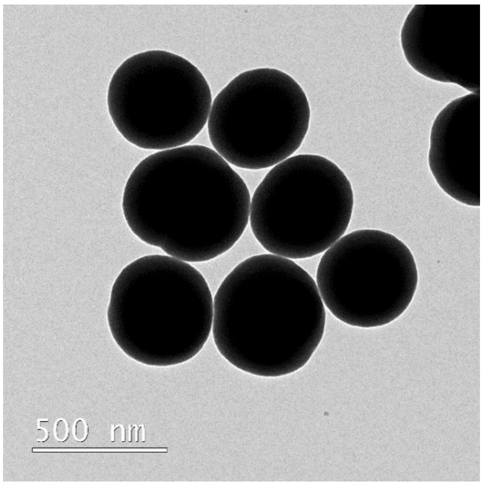

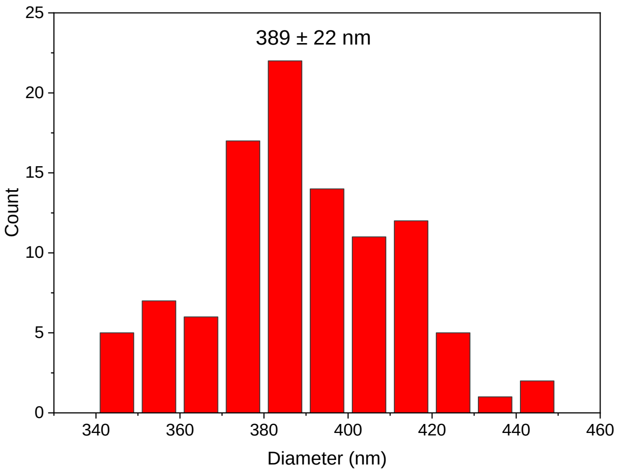

Solid state ^{29}Si NMR

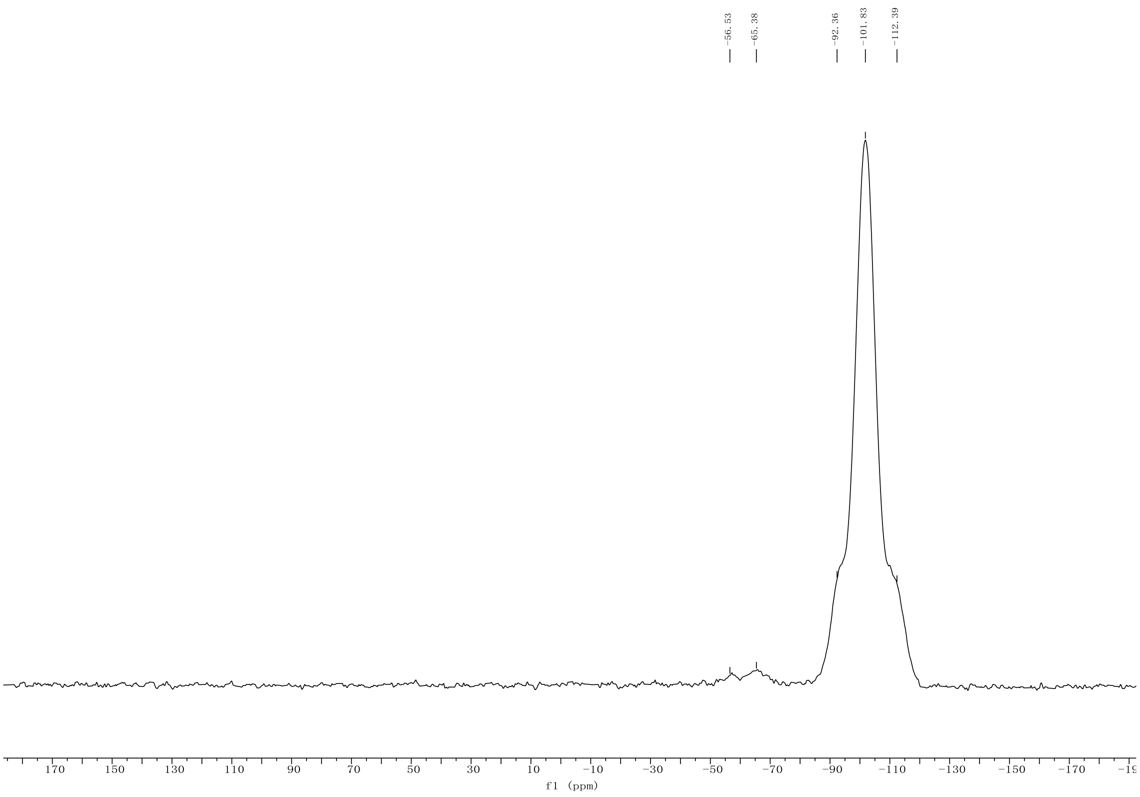

IR (ATR)

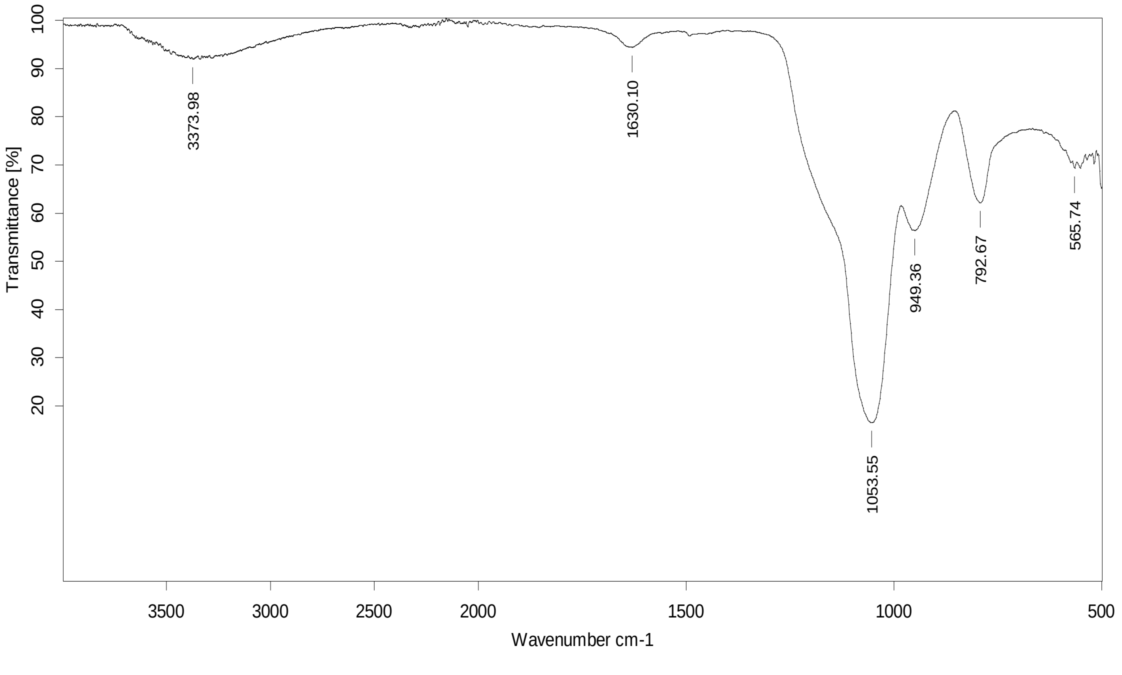

Procedure for the preparation of cotton fabrics coated with functionalized silica nanoparticles SiO_2@Norflox-Ibu (40:1)

Tetraethoxysilane (2.08 g, 10.0 mmol) and the corresponding silylated bifunctional derivative **2c** (0.25 mmol) were dissolved in absolute EtOH (25 mL). Then, an ammonium hydroxide-ethanol solution was added (6 mL of 28% $NH_3 H_2O$ in 25 mL EtOH). The mixture was magnetically stirred intensively (1400 rpm) at room temperature for 12 hours and then ultrasonicated for 30 min. Afterwards, a piece of clean cotton fabric (3 × 3 cm) was immersed in the solution and the solution was ultrasonicated for 30 min. After 30 min, the cotton fabric was removed from the solution, washed with water and ethanol for several times and dried in a vacuum oven at 50 ℃ for 12 h.

Characterization of a coated cotton fabric with SiO_2@Norflox-Ibu (TEOS: 2c (40:1))

Fabric-SiO_2@Norflox-Ibu (40:1)

SEM

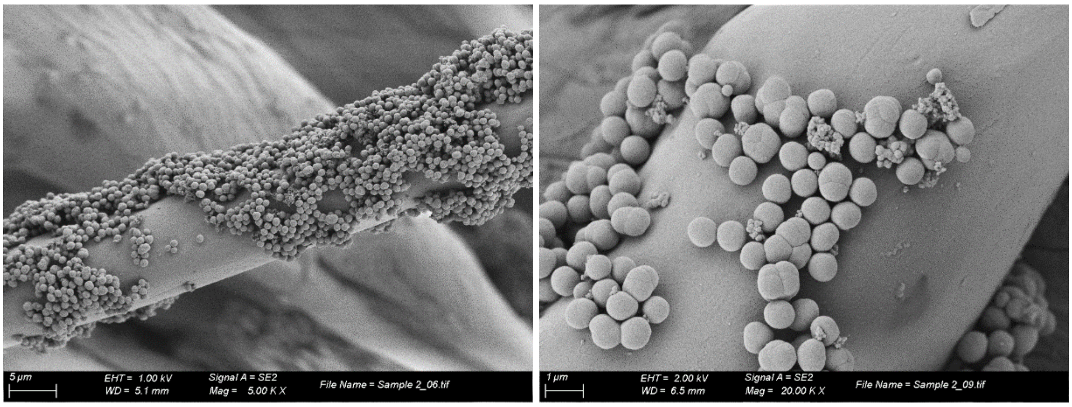

EDX mapping

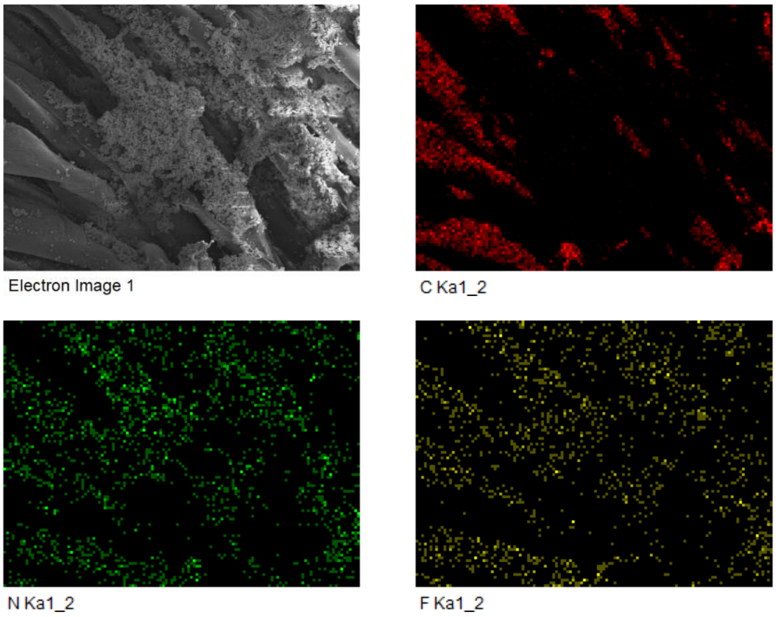

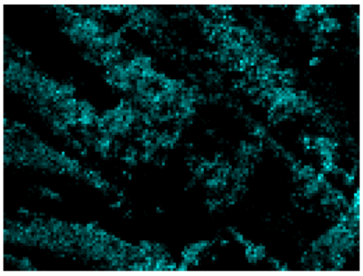

O Ka1

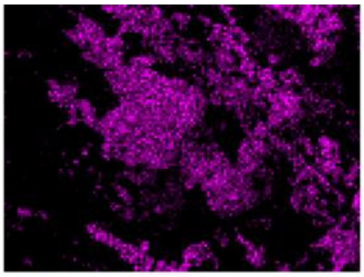

Si Ka1

EDX linear scanning

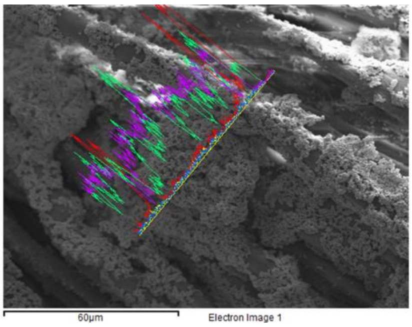

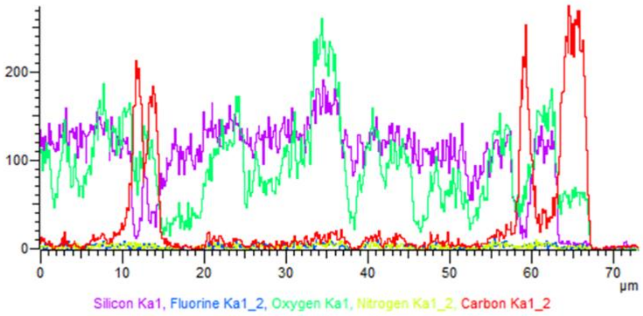

Element	App Conc.	Intensity Corrn.	Weight%	Weight% Sigma	Atomic%
C K	71.22	0.8193	24.23	0.29	34.07
N K	0.69	0.4052	0.47	0.29	0.57
O K	267.17	1.6888	44.09	0.29	46.55
F K	0.33	0.5990	0.15	0.14	0.14
Si K	121.39	1.0893	31.06	0.23	18.68
Totals			100.00		

ATR

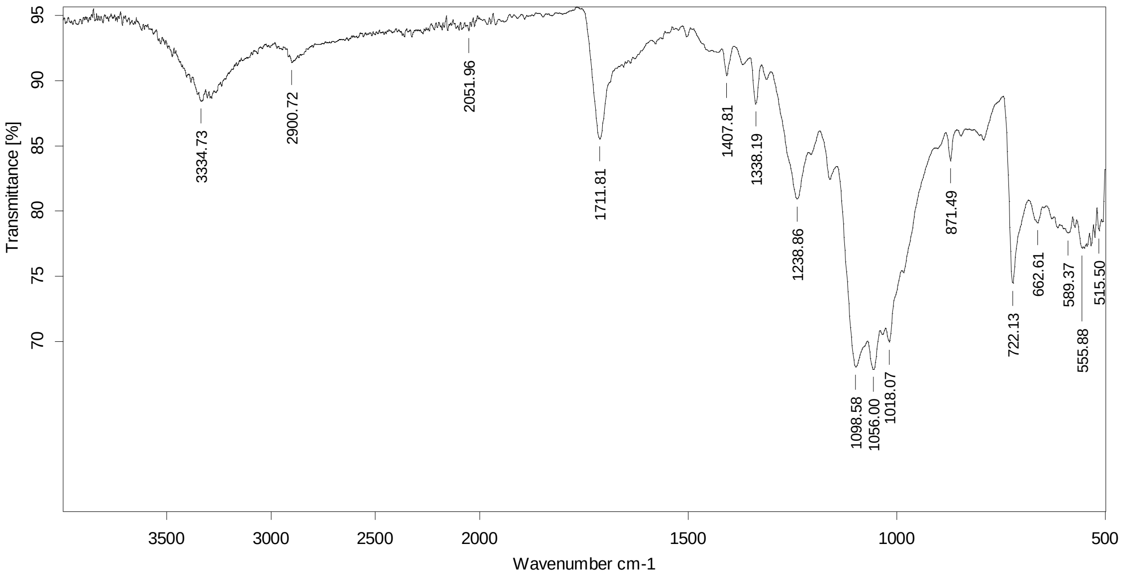

Transmittance [%]
95
90
85
80
75
70
3334.73
2900.72
2051.96
1711.81
1407.81
1338.19
1238.86
1098.58
1056.00
1018.07
871.49
722.13
662.61
589.37
555.88
515.50
3500
3000
2500
2000
1500
1000
500
Wavenumber cm-1

5. Treatment of functionalized silica nanoparticle SiO_2@Norflox-Ibu (40:1) with proteases for quantitative analysis by UV-Vis

The functionalized silica nanoparticles (20 mg) were dispersed in phosphate-buffered saline (PBS, pH 7.4) (2 mL) in an eppendorf tube, the corresponding protease (see Table 2 in the manuscript) was added and the mixture was gently stirred at 37 ℃ for the given time (orbital shaker). The concentration of protease was 0.2 mM. Then, after removal of nanoparticles by centrifugation, the supernatant was extracted with dichloromethane (10 × 3 mL). The solvent was removed under vacuum from the combined organic phases. The residue was dissolved in acetonitrile and the solution was analyzed by UV-vis (Ibuprofen 220 nm¸ Norfloxacin 285 nm).

6. Treatment of functionalized silica nanoparticle SiO_2@Norflox-Ibu (40:1) with proteases for qualitative detection of Norfloxacin

The functionalized silica nanoparticles (20 mg) were dispersed in phosphate-buffered saline (PBS) (2 mL) in an eppendorf tube, the corresponding protease was added and the mixture was gently stirred at 37 ℃ for 48 h (orbital shaker). The concentration of protease was 0.2 mM. Glacial acetic acid (2 mL) was added and the mixture was stirred for 30 min. Then after removal of nanoparticles by centrifugation, the supernatant was diluted to 10 mL and the aqueous solution was analyzed by UV-vis. The commercial norfloxacin aqueous solution (0.24 mmol/L) was prepared with the same amount of glacial acetic acid and was analyzed by UV-vis (285 nm).

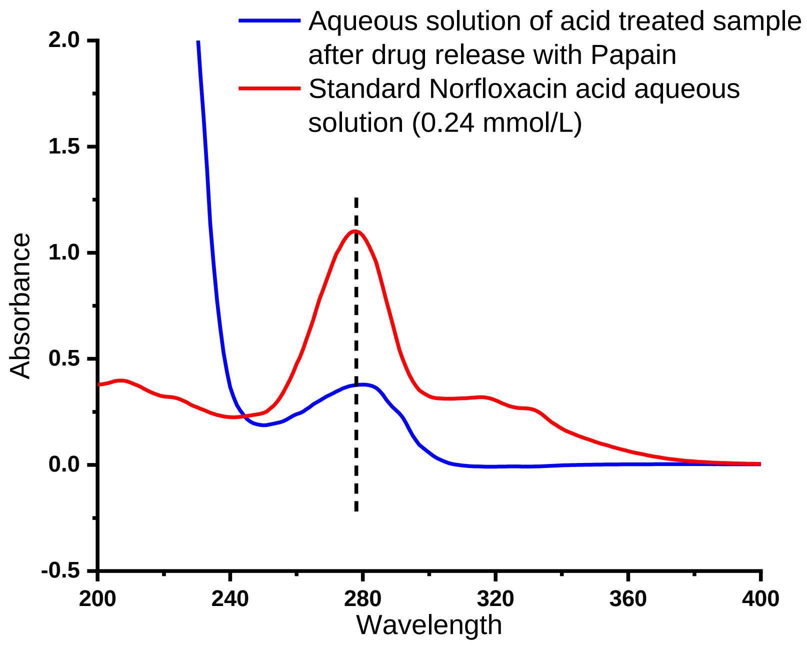

*Qualitative detection of Norfloxacin by UV-Vis after treatment of **SiO_2@Norflox-Ibu** (TEOS:**2c** 40:1) with papain for 48 h*

7. Treatment of cotton fabrics coated with functionalized silica nanoparticles with proteases

A piece of **Fabric-SiO_2@Norflox-Ibu (40:1)** (3 × 3 cm) was cut into small pieces and dispersed in phosphate-buffered saline (PBS) (5 mL) in an eppendorf tube, the corresponding protease was added and the mixture was gently stirred at 37 ℃ for 48 h (orbital shaker). The concentration of protease was 0.2 mM. After the removal of cotton fabrics, the supernatant was extracted with dichloromethane (25 × 3 mL). The solvent was removed under vacuum from the combined organic phases. The residue was dissolved in acetonitrile and the solution was analyzed by UV-vis.

8. Inhibitory tests of bacterial growth

The supernatants of papain treated **SiO_2@Norflox-Ibu** (TEOS:**2c** 40:1) and **Fabric-SiO_2@Norflox-Ibu** (TEOS:**2c** 40:1) were used to test the inhibitory effects of the released Norfloxacin on the growth of *S. Aureus.*

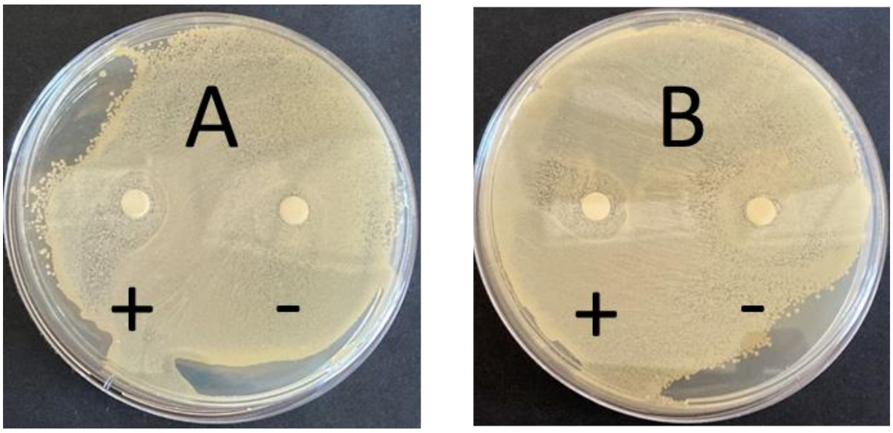

Bacterial (S. Aureus) growth is inhibited by the supernatants of the cotton fabrics (A) or nanoparticles (B) loaded with norfloxacin-ibuprofen. In each Petri plate we show the inhibition of bacterial growth after 24 h/37ºC incubation with (+) or without (-) papain. In both cases, digestion with papain enhances the release of norfloxacin resulting in wider growth inhibition halo
